# The Role of Cysteine-Rich Protein 2 in Aortic Dissection: Implications for VSMC Phenotypic Modulation—CSRP2 Impedes the Progression of Aortic Dissection

**DOI:** 10.3390/biom16081101

**Published:** 2026-07-28

**Authors:** Can Liu, Xiangyu Wang, Cheng An, Shenglin Ge, Chengxin Zhang

**Affiliations:** Department of Cardiovascular Surgery, The First Affiliated Hospital of Anhui Medical University, Wanshui Road 120, Hefei 230022, China; ahmuliucan@fy.ahmu.edu.cn (C.L.); cadiovaswxy0105@outlook.com (X.W.); ancheng613@163.com (C.A.); geshenglin@ahmu.edu.cn (S.G.)

**Keywords:** aortic dissection, vascular smooth muscle cells, phenotypic switching, cysteine-rich protein 2, p130 Crk-associated substrate

## Abstract

Aortic dissection (AD) is a severe vascular condition marked by abrupt onset, rapid progression, and heightened mortality rates. Vascular smooth muscle cells (VSMCs), the predominant cellular component of the arterial media, are essential for maintaining the structural integrity and functionality of blood vessels. Recent studies have associated Cysteine-rich protein 2 (CSRP2) with the advancement of several vascular diseases. The involvement of CSRP2 in AD progression is unclear. Aortic tissues were collected from patients for RNA sequencing and histological analysis. A mouse model of AD was created using β-aminopropionitrile monofumarate (BAPN), while VSMC phenotypic switching was induced by platelet-derived growth factor BB (PDGF-BB). Adeno-associated virus vector was used to overexpress CSRP2 in aorta. A variety of histopathological assays and biochemical analyses were applied to determine gene and protein expression patterns as well as uncover underlying molecular mechanisms. CSRP2 was significantly downregulated in both human and murine AD, and CSRP2 gene overexpression dramatically reduced BAPN-induced AD incidence and prevented animal mortality. CSRP2 could preserve a contractile VSMC phenotype, even though under PDGF-BB stimulation. Mechanistically, our findings reveal that CSRP2 directly interacts with p130 Crk-associated substrate (p130Cas; also known as BCAR1) and reduces its phosphorylation, which in turn inhibits the activation of extracellular signal-regulated kinase (ERK) signaling pathways, thereby preventing VSMC phenotypic switching induced by PDGF-BB. Our findings identify CSRP2 as a novel regulator of VSMC phenotypic modulation and a significant modulator of AD development, suggesting its potential as a target for early intervention for AD.

## 1. Introduction

Aortic dissection (AD) is characterized by the disruption of the aortic intima due to multiple factors, leading to the infiltration of blood from the rupture site into the layers of the aortic wall and resulting in separation of the aortic wall [1]. AD poses an immense risk to life, marked by arterial remodeling and associated with elevated mortality rates and serious complications. Immediate hospitalization and intervention are crucial upon diagnosis, as 80% of AD patients succumb to aortic rupture [2]. The current clinical management of AD primarily relies on surgical intervention, with limited effective pharmacotherapeutic options [3,4], underscoring the necessity to explore novel targets for AD patients.

The histopathological hallmark of AD is significant medial degeneration, characterized by considerable loss of vascular smooth muscle cells and degradation of the extracellular matrix (ECM) [5]. Vascular smooth muscle cells (VSMCs) are essential for maintaining the structural integrity and functionality of blood vessels. Under pathological stimulations, VSMCs transition from a differentiated, quiescent and contractile phenotype to a dedifferentiated, activated and synthetic state, with increased ability to proliferate and migrate [6,7,8]. Such phenotypic flexibility, or phenotypic switching, is clearly linked to progressive aortic dilation and AD pathogenesis, potentially resulting in aortic rupture [9]. Therefore, elucidating the molecular mechanisms that drive VSMC phenotypic switching in the context of AD formation is crucial for identifying novel targets and developing pharmacological strategies for AD prevention and treatment.

Cysteine-rich protein 2 (CSRP2 or CRP2), primarily expressed in VSMCs, is a member of the LIM-only CRP family, characterized by the presence of two LIM domains [10]. CSRP2 has been associated with protein assembly and reorganization of actin-based cytoskeleton [11]. Within the nucleus, CSRP2 interacts with transcription factors SRF and GATA, thereby promoting VSMC differentiation [12]. Previous research indicates that CSRP2 deficiency enhances neointima formation by promoting VSMC migration into the intima following vascular injury [13]. Moreover, CSRP2 was recognized as one of the five most significantly differentially expressed genes specific to VSMC clusters in the infrarenal abdominal aorta, with a protective role in vascular occlusion [13,14]. However, the role of CSRP2 in AD pathogenesis is unclear and warrants further study.

Herein, we attempted to investigate the potential role of CSRP2 in AD pathogenesis. Our data show that CSRP2 is a functional regulator in preserving VSMC contractile phenotype and AD development. CSRP2 exerts its function in controlling VSMC phenotypic switching primarily by modulating p130Cas/ERK signaling pathway. Our research advances the comprehension of CSRP2’s role and mechanisms in AD progression, potentially aiding in the discovery of novel targets for early intervention for this critical condition.

## 2. Materials and Methods

### 2.1. Human Aortic Samples

This study adhered to Anhui Medical University’s ethical guidelines, securing informed consent from patients and approval from the relevant review committee (protocol code 2023626). During surgical operations, human ascending aortic tissues were collected from type A aortic dissection patients who did not have connective tissue disorders such as Turner’s, Loeys–Dietz, Ehlers–Danlos, or Marfan’s syndrome. Healthy ascending aortic tissues without dissection were obtained from deceased organ donors who had no known aortic diseases. All experiments followed the principles of the Declaration of Helsinki.

### 2.2. Experimental Mouse Model of BAPN-Induced AD Formation

The C57BL/6J male mice were obtained from the Experimental Animal Centre of Anhui Medical University. All animal studies adhered strictly to the Guide for the Care and Use of Laboratory Animals (NIH guidelines). All animal study protocols received approval from the Experimental Animal Ethics Committee of Anhui Medical University (protocol code LLSC20231539).

The murine model of AD was established using similar procedures described in previous studies [15,16]. Male C57BL/6J mice, approximately three weeks old, were randomly assigned to either a regular chow diet or 0.4% β-aminopropionitrile monofumarate (BAPN, Sigma, St Louis, MO, USA) (0.4 g BAPN added to 100 g of chow diet) for up to four weeks to induce AD. In additional sets of experiments, overexpression of CSRP2 in the aorta was achieved by tail vein injection of control (AAV9-SCR) or CSRP2 (AAV9-CSRP2) adeno-associated virus serotype 9 (AAV9) vector (Hanheng Biotechnology, Shanghai, China) into 3-week-old male C57BL/6J mice. One week later, mice were subjected to similar procedures as previously described to induce AD formation. Body weight and blood pressure were measured weekly. A non-invasive tail-cuff system was used at the same time of day to minimize circadian variation of blood pressure. Each session consisted of 5 consecutive recordings, of which the mean value was used for analysis. Mice were allowed to recover for 72 h after tail-vein injection before the first post-operative measurement to avoid acute hemodynamic perturbations from the procedure. Mice with sudden death during the study were immediately dissected to determine whether death was caused by aortic dissection. At the end of the protocol, the remaining surviving mice were euthanized through deep anesthesia using 100% O2/5% isoflurane, followed by decapitation. Aortic tissues were collected for subsequent histopathological analysis.

### 2.3. Histological Analysis

Aortic specimens underwent fixation in 4% paraformaldehyde overnight, followed by dehydration, paraffin embedding, and sectioning into 4 µm thick slices. The sections were stained with hematoxylin and eosin (H&E) or elastin Verhoeff-van-Giessen (EVG) and assessed using an optical microscope. A blinded observer performed a qualitative evaluation of elastin integrity through the analysis of digital images, employing a semiquantitative grading system to guarantee an unbiased assessment. The grading criteria for elastin preservation were defined as follows: grade 1 indicates intact elastin fibers with no degradation; grade 2 for mild interruptions in the elastic laminae; grade 3 denotes multiple disruptions in the elastic laminae; and grade 4 signifies severe fragmentation or aortic rupture of the elastic laminae.

### 2.4. Immunohistochemistry (IHC) and Immunofluorescence (IF) Techniques

For IHC staining, paraffin slices were deparaffinized, placed in boiling Tris-EDTA antigen retrieval buffer for antigen retrieval, and treated with hydrogen peroxide to inhibit endogenous peroxidase activity. After a blocking phase with 5% BSA, diluted primary antibodies were administered to the sections and incubated overnight at 4 °C. The sections were then treated with an HRP-conjugated antibody and incubated for 30 min at ambient temperature. The tissues were counterstained with hematoxylin and DAB and then examined under a light microscope.

For immunofluorescence staining, sections underwent antigen retrieval, were blocked with goat serum, and incubated overnight at 4 °C with either a primary antibody or an IgG control. The sections were subsequently incubated with the relevant secondary antibodies and stained using DAPI. Fluorescence was observed and recorded using a fluorescence microscope.

Semi-quantitative analysis was performed using ImageJ (v1.53e). For IHC, the Mean Optical Density (MOD = Integrated Density/Area) was calculated via the ‘Color Deconvolution’ plugin to isolate specific signals from the background. For IF, the mean fluorescence intensity of the target protein was measured within the defined vessel wall region of interest (ROI), with background subtraction applied. For both modalities, at least three random high-power fields per section were analyzed in a blinded manner, and data were normalized to controls.

### 2.5. Cell Culture and Transfection

MOVAS cells (murine vascular smooth muscle) were obtained from the Cell Bank of CEMCS (Chinese Academy of Sciences Center for Excellence in Molecular Cell Science, Shanghai, China). These cells were cultured in Dulbecco’s modified Eagle medium (DMEM, GIBCO, Carlsbad, CA, USA) supplemented with 10% fetal bovine serum (FBS, ExCell Bio, Shanghai, China) at 37 °C in an incubator with 5% CO_2_. CSRP2 siRNA and its negative control were sourced from GenePharma in Shanghai, China. The control and pcDNA-CSRP2 vector from HanBio Technology (Shanghai, China) were utilized for the overexpression of CSRP2 in MOVAS cells. Upon achieving 60–70% confluence, MOVAS cells in 6-well plates were transfected with siRNA reagent or plasmid mixture using Lipofectamine 2000 (Invitrogen, Waltham, MA, USA) following the manufacturer’s instructions. Cells underwent serum starvation for 24 h and were subsequently treated with platelet-derived growth factor subunit BB (PDGF-BB, 10 ng/mL) for an additional 24 h to induce VSMC phenotypic switching.

### 2.6. Western Blotting

Total proteins were extracted from cells or aortas and subsequently analyzed via SDS polyacrylamide gel electrophoresis. Proteins were transferred to PVDF membranes (Merck Millipore, Billerica, MA, USA) and blocked with 5% skimmed milk for 2 h, followed by overnight incubation with primary antibodies at 4 °C. The membranes were then incubated with appropriate secondary antibodies at room temperature for two hours. Protein bands were visualized with a chemiluminescence kit (ZEN-BIOSCIENCE, Chengdu, China) and quantified using Image J (version 1.53, Bethesda, MD, USA). Original western blot images can be found in Supplementary Figure S4.

Antibodies against CSRP2 (PH0995, 1:1000), myosin heavy chain 11 (MYH11, T58529, 1:2000), α-smooth muscle actin (α-SMA, TA1032, 1:1000), Osteopontin (T55333, 1:2000), Calponin (CNN1, T55204, 1:1000), smooth muscle protein 22-α (SM22-α, PC2178, 1:2000), β-actin (TA7018, 1:500), p130Cas (PA6071, 1:1000), Phospho-p130Cas (Tyr410, TA3350, 1:1000), ERK1/2 (T40071, 1:1000), and Phospho-ERK1/2 (TA1015, 1:1000) were purchased from Abmart (Shanghai, China). Antibodies against CSRP2 (Rabbit, 10892-2-AP) and BCAR1 (Rabbit, 30887-1-AP) were purchased from Proteintech (Wuhan, China).

### 2.7. Real-Time Quantitative PCR (RT-qPCR)

Trizol reagent was utilized for the extraction of total RNA from cells and tissues. The Prime Script RT Master Mix Kit (Takara, Kyoto, Japan) was utilized for cDNA synthesis. RT-qPCR was conducted using the T100 Real-Time PCR system (Bio-Rad, Hercules, CA, USA) with the SYBR Green Kit (Takara, Kyoto, Japan). mRNA levels were quantified using the 2^−ΔΔCt^ method. Primer sequences used in this study are included in Table 1.

### 2.8. Cell Proliferation Assay

Cells were seeded in 96-well plates and cultured for 24 h for the CCK-8 assay. CCK-8 solution (Beyotime, Shanghai, China) was introduced into each well and incubated for one hour. Absorbance at 450 nm was recorded with a microplate reader (Bio-Rad, Hercules, CA, USA).

EdU staining was performed using the EdU Cell Proliferation Kit (RiboBio, Guangzhou, China) following the manufacturer’s instructions. Cells were treated with a 50 µM EdU solution for 2 h, followed by staining with Apollo Dye and Hoechst solution, and fixed using 4% paraformaldehyde. Cells were then observed and captured utilizing a fluorescence microscope.

### 2.9. Wound Healing Assay

Cells were plated in a 6-well plate and incubated for 24 h. Wounds were created using 200 μL pipettes, followed by cell culture in DMEM supplemented with 2% FBS. Wound closure was assessed at 0, 12, and 24 h, respectively. The wound area was standardized to the 0 h measurement to evaluate cell migration capability.

### 2.10. Transwell Assay

A total of 2 × 10^5^ cells were placed in the upper chamber of the transwell (8-μm-pore, Corning, Tewksbury, MA, USA). Then, 200 μL of DMEM without FBS was introduced into the upper chamber, while 500 μL of DMEM containing 10 ng/mL PDGF-BB was added in the lower compartment. After a 24 h migration period, cells were fixed with 4% paraformaldehyde and stained with 0.1% crystal violet for 15 min. Ultimately, five visual fields within each membrane were randomly selected for imaging and quantification via microscopy.

### 2.11. Co-Immunoprecipitation (Co-IP) Assay

Cells were collected and lysed in cold RIPA buffer and then centrifuged at 1400 g for 15 min. Cell lysates were incubated with control IgG and protein A/G agarose beads to minimize non-specific binding, followed by incubation with either IgG control or anti-CSRP2 (or anti-p130Cas) antibody overnight at 4 °C with rotation. Fresh protein A/G agarose beads were added into the antibody-bound lysate, allowing the antibody–protein complex to bind to the beads. The beads were repeatedly washed with buffer, after which the protein complexes were eluted, mixed with SDS-PAGE sample buffer, and heated for protein denaturation. The immunoprecipitated proteins underwent SDS-PAGE separation followed by Western blot analysis as previously outlined.

### 2.12. Construction of Wild-Type and Phosphor-Dead P130Cas and ERK1/2 Inhibition

The p130Cas-WT is a wild-type p130Cas plasmid created by inserting the full length of the coding sequence of the BCAR1 gene (NM_001198839) into the PCDNA3.1 vector. The p130Cas phospho-dead mutant, p130Cas-F15, is a mutant plasmid vector created by substituting all 15 tyrosines in the p130Cas substrate domain with phenylalanine. Both p130Cas-WT and p130Cas-F15 plasmids were constructed by Hanheng Biotechnology and confirmed by DNA sequencing. U0126 (Cell Signaling, Danvers, MA, USA), a potent and selective MAPK kinase and ERK1/2 inhibitor, was utilized to block ERK1/2 signaling in MOVAS cells.

### 2.13. RNA Sequencing and Data Analysis

RNA sequencing and data analysis were performed as described previously with minor modifications [17,18]. Total RNA was extracted using TRI Reagent^®^ solution (Sigma, St Louis, MO, USA), and mRNA was subsequently purified from the total RNA with oligo (dT) magnetic beads. RNA quality control and cDNA library preparation were conducted at Personalbio Technology (Shanghai, China). The cDNA library quality was evaluated with the Agilent 2100 Bioanalyzer and subsequently sequenced using the NovaSeq 6000 (Illumina, San Diego, CA, USA). Sequencing reads, termed raw reads, were obtained from the original image data generated by NovaSeq through base calling. An in-house NGS pipeline was employed for the analysis of sequencing data. Raw sequence reads were trimmed to eliminate adapter sequences and low-quality nucleotides (error rate < 0.05) at the ends. Sequence reads under 50 nucleotides were excluded after trimming. The remaining sequence reads were aligned to the reference genome, using hg38 for human and GRCm38/mm10 for mouse in AD RNA sequencing. Gene hit counts were quantified, and RPKM values were computed. Genes with *p*-values below 0.05, determined via the Wald test, were identified as differentially expressed for each comparison. Differentially expressed genes (DEGs) were analyzed using the ‘Express Analysis’ feature on Metascape (https://metascape.org/gp/index.html#/main/step1, accessed on 16 June 2024). The fold changes across various comparisons were log2 transformed to center the data around zero, while the *p*-values were transformed using −log10 for volcano plot analysis.

### 2.14. Statistical Analysis

The data for this study were obtained from at least three independent biological replicates, reported as $\overset{-}{x}$ ± SD. Statistical analyses were conducted using GraphPad (version 9.0, San Diego, CA, USA). The Shapiro–Wilk normality test and an F-test were employed to assess the normality and homogeneity of variance of the datasets, respectively. Comparisons of mean values between two independent groups were conducted using Student’s *t*-test, while comparisons among multiple groups were executed using one-way ANOVA. Pairwise comparisons among groups were conducted using the Least Significant Difference test, as our analyses focused on specific, pre-planned comparisons rather than all possible pairwise combinations. *p* < 0.05 is regarded as statistically significant.

## 3. Results

### 3.1. CSRP2 Is Downregulated in Human and Murine AD

**Figure 1:**
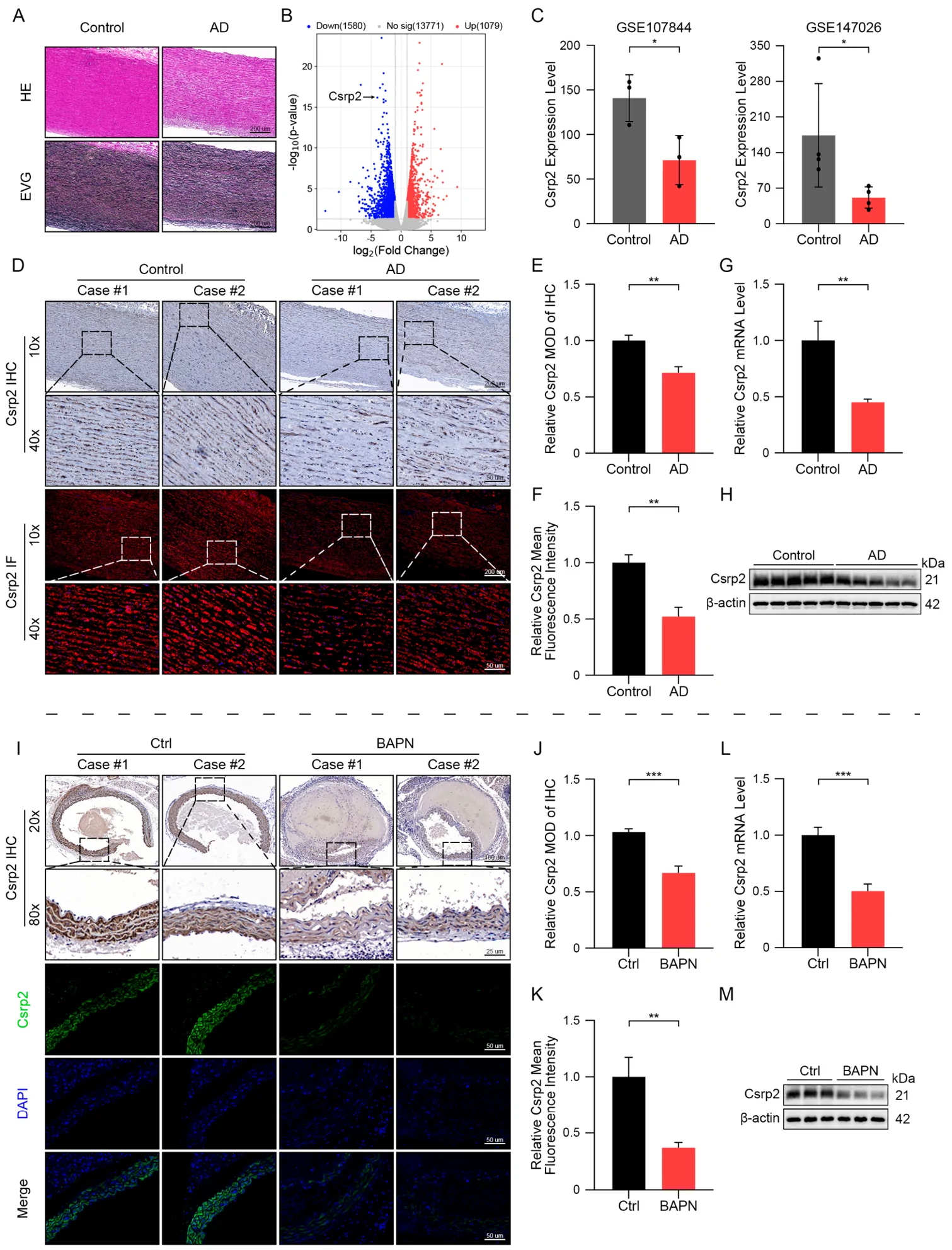
CSRP2 is downregulated in human ((**A**–**H**), *n* = 5 patients per group) and murine ((**I**–**M**), *n*= 3–5 mice per group) AD. (**A**) Representative H&E and EVG staining of human aortic tissues. Scale bar = 200 μm. (**B**) Volcano plot showing differentially expressed genes between control and AD patients identified by RNA-sequencing analysis. (**C**) Relative CSRP2 mRNA levels of healthy controls and AD patients in GSE107844 or GSE147026 datasets. (**D**) Representative immunohistochemistry (IHC) and immunofluorescence (IF) staining for CSRP2 in human aortic tissues. Scale bar = 200 or 50 μm. (**E**,**F**) Relative expression levels of CSRP2 in human aortic tissues were compared and quantified by mean optical density (MOD for IHC staining, (**E**)) or mean fluorescence intensity (for IF staining, (**F**)). (**G**) RT-qPCR was performed to detect CSRP2 mRNA expression in human aortic tissues. (**H**) Western blot was performed to detect CSRP2 protein expression in human aortic tissues. Original western blot images see supplementary Figure S4. (**I**) Representative IHC and IF staining for CSRP2 in control- and BAPN-treated mouse aortic tissues. Scale bar = 100, 50 or 25 μm. (**J**,**K**) Relative expression level of CSRP2 in control- or BAPN-treated mouse aortic tissues were compared and quantified by MOD ((**J**), IHC) or mean fluorescence intensity ((**K**), IF). (**L**) RT-qPCR was performed to detect CSRP2 mRNA expression in control- and BAPN-treated mouse aortic tissues. (**M**) Western blot was performed to detect CSRP2 protein expression in control- and BAPN-treated mouse aortic tissues. Data are shown as mean ± standard deviation, * *p* < 0.05, ** *p* < 0.01, *** *p* < 0.001 versus control group.

Five pairs of clinical samples from AD patients or healthy individuals were collected in the clinical biobank of the First Affiliated Hospital of Anhui Medical University. Loss of VSMCs and degradation of elastic fibers were clearly evident through H&E and EVG staining in human AD tissues, but not in control healthy aortic tissues (Figure 1A). RNA sequencing and bioinformatics analyses were performed to identify potential regulatory genes in human AD. We found 6048 DEGs between normal healthy human aorta and AD, while 2435 and 3613 genes were upregulated and downregulated in human AD, respectively. Pathway enrichment analysis of upregulated DEGs showed that a variety of signaling pathways such as ‘Cytokine signaling in immune cells’, ‘Neutrophil degranulation’, ‘Cellular response to cytokine stimulus’, ‘Regulation of apoptotic signaling pathway’, ‘Regulation of proteolysis’, ‘Transcriptional regulation by TP53′, ‘Regulation of protein stability’, ‘Positive regulation of programmed cell death’, ‘Inflammatory response’, and ‘positive cell migration and proliferation’ were significantly enriched in human AD (Supplementary Material online, Figure S1A), indicating that these signaling pathways are apparently activated in human AD. On the other hand, pathway enrichment analysis of downregulated DEGs showed multiple signaling pathways associated with ‘Actin filament-based processes’, ‘Muscle cell differentiation’, ‘Circulatory system processes’, ‘Muscle contraction’, ‘Smooth muscle contraction’, and ‘Extracellular matrix organization’ were significantly enriched in human AD (Supplementary Material online, Figure S1B), suggesting that these signaling pathways are significantly inhibited in human AD. CSRP2, known as a VSMC differentiation regulator, was notably among the most significantly downregulated genes in human AD (Figure 1B). To further ascertain the involvement of CSRP2 in aortic dissection progression, additional analysis was conducted in two AD cohorts obtained from the Gene Expression Omnibus databases (GEO, https://www.ncbi.nlm.nih.gov/geo/, accessed on 17 July 2024). The GSE107844 dataset contains gene expression profiles from three patients with thoracic aortic dissection and three healthy controls, while the GSE147026 dataset includes profiles from four patients with acute aortic dissection and four healthy controls. In these two independent cohorts, a notable decrease in CSRP2 mRNA levels was observed in AD patients (Figure 1C). IHC and IF staining were utilized to assess the expression level of CSRP2 in human aortic tissues. CSRP2 was primarily found in the cytoplasm of VSMCs, with notably lower expression in human AD tissues compared to controls (Figure 1D–F). RT-qPCR and WB analyses further supported a downregulation of CSRP2 expression in AD patients compared to the control group (Figure 1G,H).

To further validate whether above observations are applicable in murine AD, a BAPN-induced AD murine model was established in C57BL/6J mice as previously reported [15,16]. IHC and IF analyses revealed significantly decreased CSRP2 expression in aortic tissue sections of BAPN-treated mice compared to controls (Figure 1I–K). Similarly, RT-qPCR and WB analyses revealed a decreased expression of CSRP2 in the aorta of BAPN-treated mice (Figure 1L,M). These findings have collectively shown that CSRP2 is significantly decreased in both human and murine AD, suggesting a protective factor of CSRP2 in AD.

### 3.2. CSRP2 Regulates AD Development and Preserves VSMC Contractile Phenotype In Vivo

**Figure 2:**
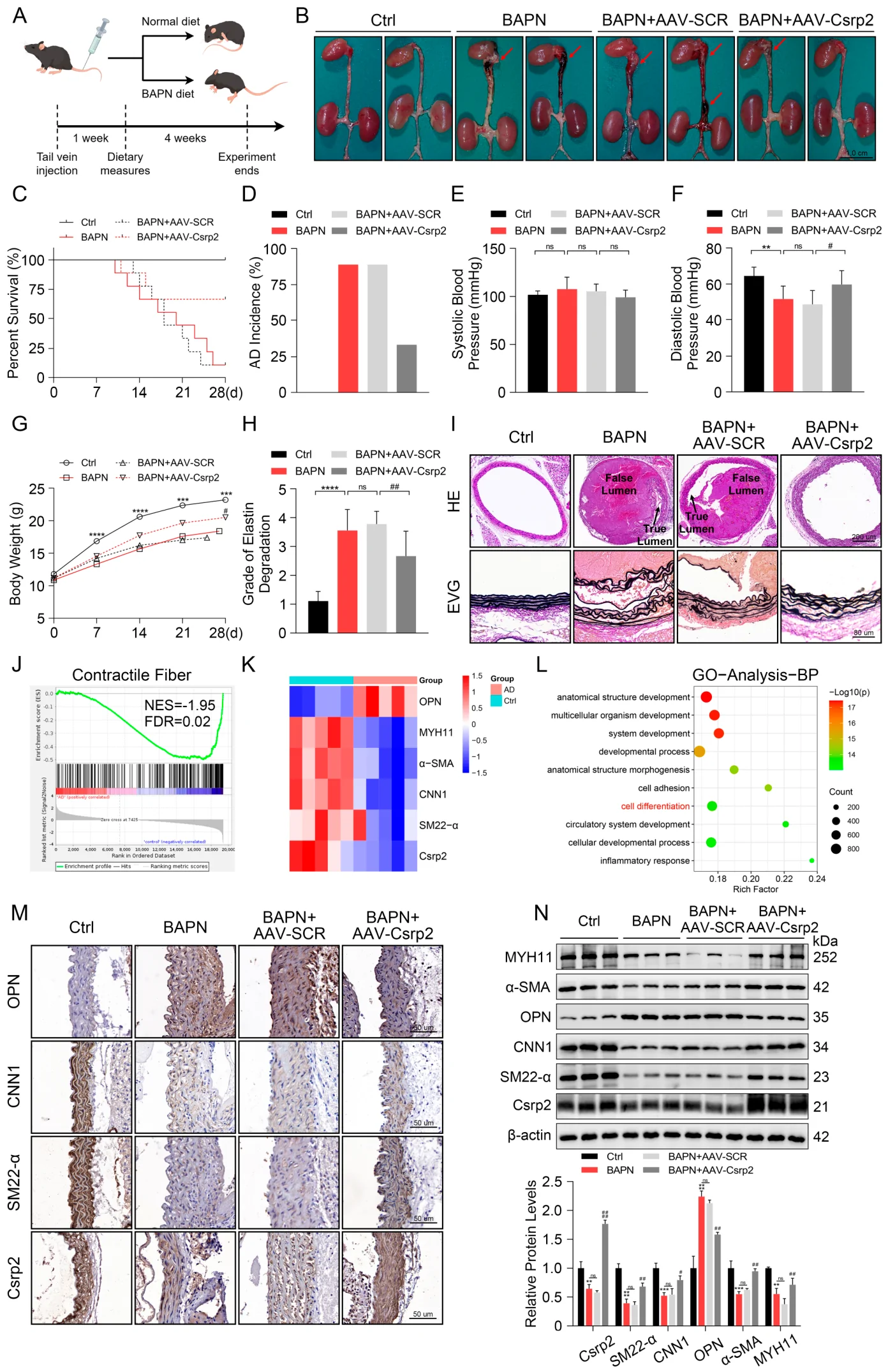
CSRP2 overexpression protects against BAPN-induced AD formation and preserves VSMC contractile phenotype in mice. (**A**) Schematic protocol: scrambled or CSRP2 overexpression AAV9 vector was randomly injected into 3-week-old male mice via tail vein. One week later, mice were fed with a regular chow diet or 0.4% BAPN diet for up to 4 weeks. Thoracic aortic tissues were collected and subjected to a variety of analysis. (**B**) Representative macroscopic images of mouse aortas with indicated treatments. Scale bar = 1.0 cm. (**C**) Kaplan–Meier survival curves for indicated groups. (**D**–**F**) AD incidence (**D**), systolic (**E**) and diastolic (**F**) blood pressures in mice with indicated treatment. (**G**) Line graph showing weekly body weight changes of mice with indicated treatments. (**H**) Elastin degradation in aortic wall. (**I**) Representative H&E and EVG staining of mouse aortic tissues. Scale bar = 200 or 80 μm. (**J**,**K**) RNA sequencing analysis of healthy and dissected human thoracic aortic tissues. (**J**) GSEA analysis showing negative regulation of ‘Contractile fiber’ pathway in clinical AD samples. NES, normalized enrichment score. FDR, false discovery rate. (**K**) Heatmap showing gene expression patents of CSRP2, contractile and synthetic VSMC markers in human control and AD samples. Representative down- (blue box) and upregulated (red box) genes are listed. (**L**) Gene Ontology analysis showing enriched biological processes (BP) in CSRP2 overexpressed mouse aortic tissues. (**M**) Representative immunostaining for OPN, CNN1, SM22-α and CSRP2 in mouse aortic tissues. Scale bar = 50 μm. (**N**) Western blot and quantitative analysis showing protein expression alterations in mouse aortic tissues with indicated treatments. Original western blot images see supplementary Figure S4. Data are shown as mean ± standard deviation, ** *p* < 0.01, *** *p* < 0.001, **** *p* < 0.0001 versus Ctrl group, ^#^ *p* < 0.05, ^##^ *p* < 0.01, ^####^ *p* < 0.0001 versus BAPN + AAV-SCR group, *n* = 5–9.

We utilized AAV9 vectors for in vivo overexpression of CSRP2 to investigate its role in AD pathogenesis, as detailed in Figure 2A. The mice were euthanized five weeks later, and the aortic tissues were collected for subsequent analysis (Figure 2B–I). BAPN treatment provoked a remarkable high mortality and AD incidence (Figure 2C,D), both were markedly mitigated by CSRP2 overexpression (Figure 2B–D). Of note, BAPN treatment reduced diastolic blood pressure, with limited impact on systolic blood pressure (Figure 2E,F), suggesting increased aortic stiffness. Additionally, BAPN treatment decreased body weight gains over the whole duration (Figure 2G). Likewise, these effects were counterbalanced by CSRP2 overexpression (Figure 2E–G). H&E and EVG staining were utilized to examine pathological features. BAPN treatment resulted in tearing of the aortic wall, formation of thrombi within false lumens, and depletion of elastic fibers. All these changes closely resembled those observed in human AD aortic tissues. Importantly, all of the observed pathological alterations were apparently mitigated by CSRP2 overexpression (Figure 2H,I). These findings have collectively indicated a protective effect of CSRP2 overexpression on BAPN-induced AD formation.

RNA sequencing was performed to examine differential gene expression in aortic tissues from both healthy individuals and those with aortic disease. Gene set enrichment analysis (GSEA) of RNA sequencing data revealed a notable downregulation of contractile fiber-related genes in AD patients compared to healthy individuals (Figure 2J). Heatmap revealed reduced expression of CSRP2 and VSMC contractile genes such as SM22-α, CNN1, α-SMA, and MYH11, alongside an increased expression of OPN (osteopontin, a synthetic VSMC marker) in human AD samples (Figure 2K), suggesting a potential role of CSRP2 in VSMC phenotypic switching (or VSMC de-differentiation) in the context of AD. Gene ontology (GO) analysis of RNA sequencing data from mouse aortic tissues with or without CSRP2 overexpression revealed that biological processes linked to multiple developmental processes including circulatory system, cell adhesion, inflammatory response, and cell differentiation were significantly enriched in CSRP2-overexpressing aortic tissues (Figure 2L), suggesting a role for CSRP2 in these biological processes, particularly in circulatory system development and VSMC differentiation in the context of AD.

Additional IHC (Figure 2M, Supplementary Material online, Figure S2A–D) and IF (Supplementary Material online, Figure S2E–G) staining of mouse aortic tissues revealed that BAPN treatment significantly promoted a synthetic VSMC phenotype, indicated by elevated OPN expression and reduced VSMC contractile markers (CNN1 and SM22-α), such phenotypic switching was counterbalanced by CSRP2 overexpression. IF staining demonstrated the colocalization of α-SMA and CSRP2 in aortic tissues (Supplementary Material online, Figure S2E). Additionally, both IHC and IF analyses confirmed elevated expression of CSRP2 in mice administered the AAV9-CSRP2 vector (Supplementary Material online, Figure S2D,G). Western blot analysis consistently suggested reduced expression levels of VSMC contractile markers (SM22-α, CNN1, α-SMA, MYH11) and CSRP2, alongside an elevated level of the synthetic marker OPN after BAPN treatment. However, these effects were reversed by CSRP2 overexpression (Figure 2N). Taken together, these data suggest that CSRP2 overexpression exerts a protective role against AD development by preventing BAPN-induced VSMC phenotypic switching.

### 3.3. CSRP2 Regulates VSMC Proliferation, Migration and Phenotypic Switching

**Figure 3:**
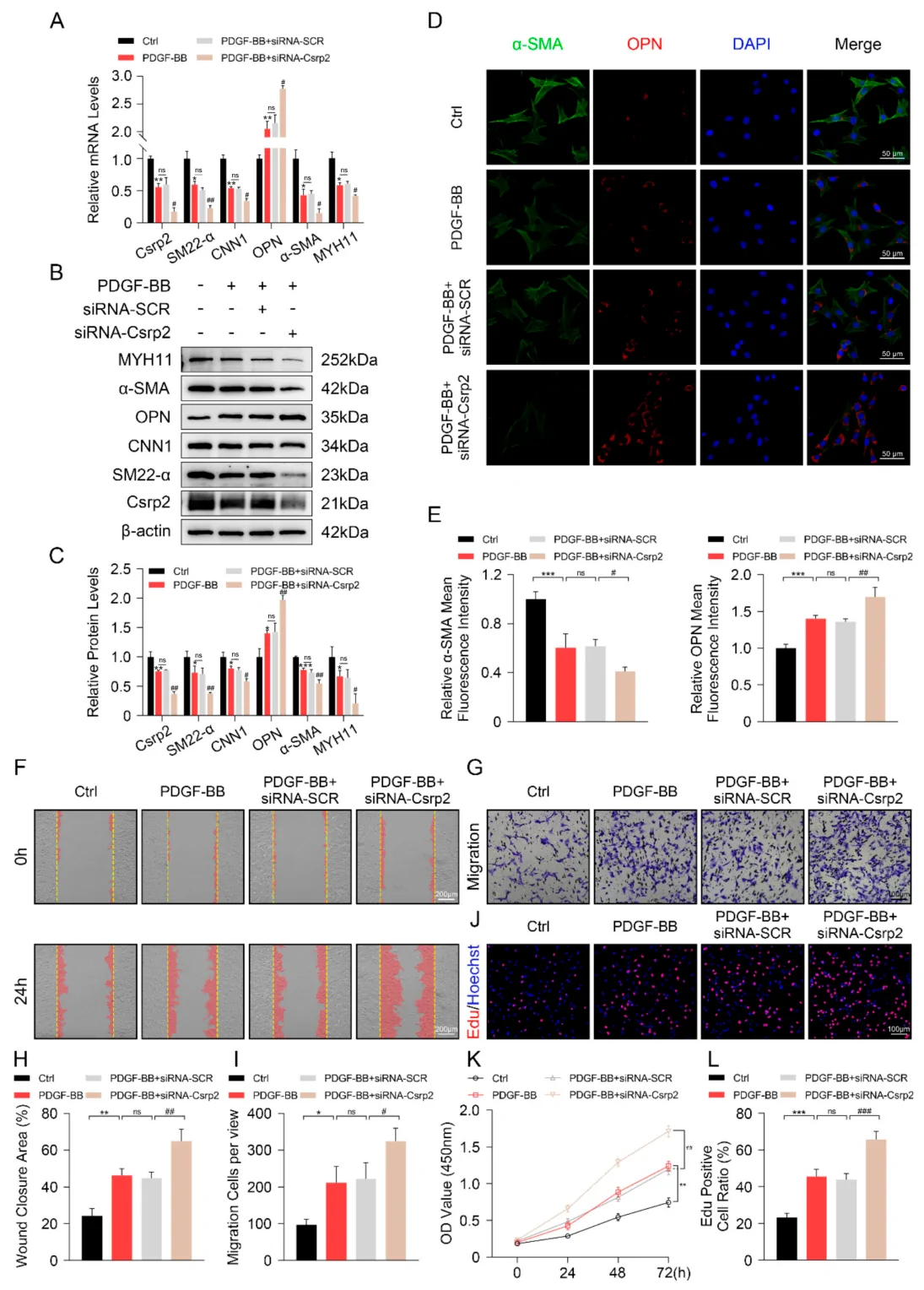
CSRP2 inhibition enhances PDGF-BB-induced VSMC phenotypic switching in vitro. MOVAS cells transfected with siRNA-Csrp2 or scrambled vectors were subjected to serum starvation for 24 h. Serum-starved cells were incubated with vehicle control or 10 ng/mL PDGF-BB for additional 24 h, followed by different analysis. (**A**) RT–qPCR analysis of SM22-α, CNN1, OPN, α-SMA and MYH11 in MOVAS cells with indicated treatments. (**B**,**C**) Western blot analysis and quantification of SM22-α, CNN1, OPN, α-SMA and MYH11 in MOVAS cells with indicated treatments. Original western blot images see supplementary Figure S4. (**D**,**E**) Immunofluorescence staining and mean fluorescence intensity of α-SMA and OPN in MOVAS cells with indicated treatments. Scale bar = 50 μm. (**F**–**I**) MOVAS cell migration was measured by wound healing (**F**,**H**) and transwell (**G**,**I**) assays, respectively. Scale bar = 200 or 100 μm. (**J**–**L**) MOVAS cell proliferation was determined with EdU staining (**J**,**L**) and CCK-8 assay (**K**), respectively. Scale bar = 100 μm. Data are shown as mean ± standard deviation, * *p* < 0.05, ** *p* < 0.01, *** *p* < 0.001 versus Ctrl, ^#^ *p* < 0.05, ^##^ *p* < 0.01, ^###^ *p* < 0.001 versus PDGF-BB + siRNA-SCR, ‘ns’ indicates non-significant difference, *n* = 3.

**Figure 4:**
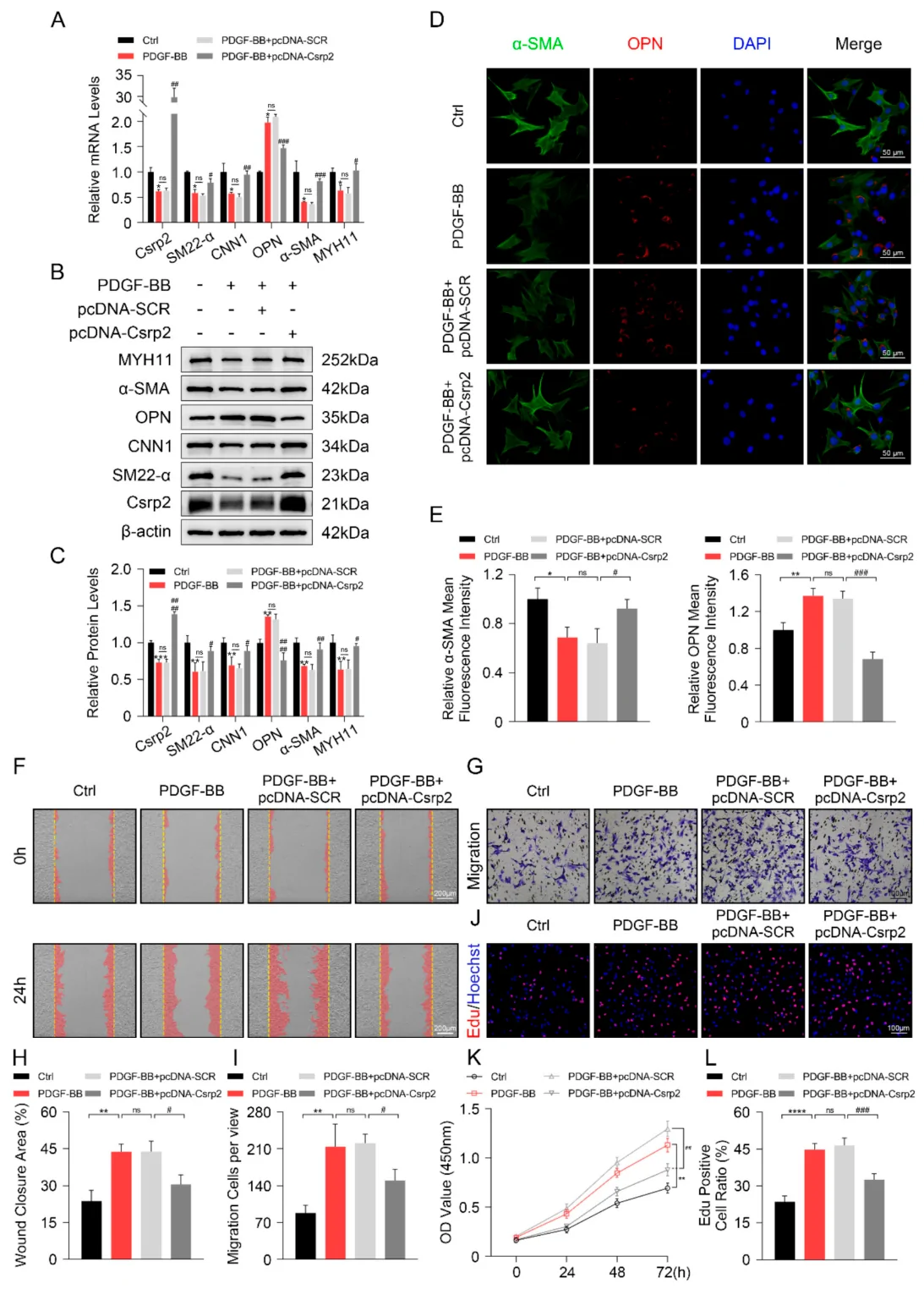
CSRP2 overexpression reverses PDGF-BB-induced VSMC phenotypic switching in vitro. MOVAS cells transfected with pcDNA-CSRP2 or scrambled vectors were subjected to serum starvation for 24 h. Serum-starved cells were incubated with vehicle control or 10 ng/mL PDGF-BB for additional 24 h, followed by different analysis. (**A**) RT–qPCR analysis of SM22-α, CNN1, OPN, α-SMA and MYH11 in MOVAS cells with indicated treatments. (**B**,**C**) Western blot analysis and quantification of SM22-α, CNN1, OPN, α-SMA and MYH11 in MOVAS cells with indicated treatments. Original western blot images see supplementary Figure S4. (**D**,**E**) Immunofluorescence staining and mean fluorescence intensity of α-SMA and OPN in MOVAS cells with indicated treatments. Scale bar = 50 μm. (**F**–**I**) MOVAS cell migration was measured with wound healing (**F**,**H**) and transwell (**G**,**I**) assays, respectively. Scale bar = 200 or 100 μm. (**J**–**L**) MOVAS cell proliferation was determined by EdU staining (**J**,**L**) and CCK-8 assay (**K**), respectively. Scale bar = 100 μm. Data are shown as mean ± standard deviation, * *p* < 0.05, ** *p* < 0.01, *** *p* < 0.001, **** *p* < 0.0001 versus Ctrl, ^#^ *p* < 0.05, ^##^ *p* < 0.01, ^###^ *p* < 0.001, ^####^ *p* < 0.0001 versus PDGF-BB + pcDNA-SCR, ‘ns’ indicates non-significant difference, *n* = 3.

To elucidate the functional role of CSRP2 in VSMCs, siRNA (Figure 3) and pcDNA vectors (Figure 4) were utilized to silence and overexpress CSRP2 in the MOVAS cells, respectively. PDGF-BB stimulation was used to induce VSMC phenotypic switching. RT-qPCR (Figure 3A) and Western blot (Figure 3B,C) analyses showed that PDGF-BB treatment significantly decreased CSRP2 expression level, which was further downregulated by siRNA-CSRP2 in MOVAS cells. As expected, PDGF-BB treatment induced MOVAS transition from a contractile phenotype to synthetic phenotype, as evidenced by decreased expression levels of contractile VSMC markers (SM22-α, CNN1, α-SMA, and MYH11) but increased expression levels of synthetic VSMC marker OPN (Figure 3A–C). Importantly, PDGF-BB-induced VSMC phenotypic switching was further exacerbated by CSRP2 inhibition (Figure 3A–C). Such observations were further confirmed using IF staining analysis (Figure 3D,E).

While contractile VSMCs display limited migration and proliferation, synthetic VSMCs exhibit increased migration and proliferation [19]. Indeed, PDGF-BB treatment significantly increased MOVAS cell migration and proliferation, which were further enhanced by the silencing of CSRP2, as supported in wound healing assays (Figure 3F and Figure 3H), transwell migration analysis (Figure 3G and Figure 3I), EdU staining (Figure 3J and Figure 3L), and CCK-8 assay (Figure 3K), respectively.

Conversely, we observed an opposing effect with CSRP2 overexpression (Figure 4). Specifically, we found that CSRP2 overexpression could reverse PDGF-BB-induced VSMC phenotypic switching from contractile to synthetic cells, as confirmed by gene expression (Figure 4A), protein alterations (Figure 4B–E), and cellular migration (Figure 4F–I) and proliferation (Figure 4J–L). The results have collectively indicated a regulatory role for CSRP2 in mitigating PDGF-BB-induced VSMC phenotypic switching.

### 3.4. CSRP2 Modulates p130Cas Phosphorylation and ERK1/2 Signaling Pathway

**Figure 5:**
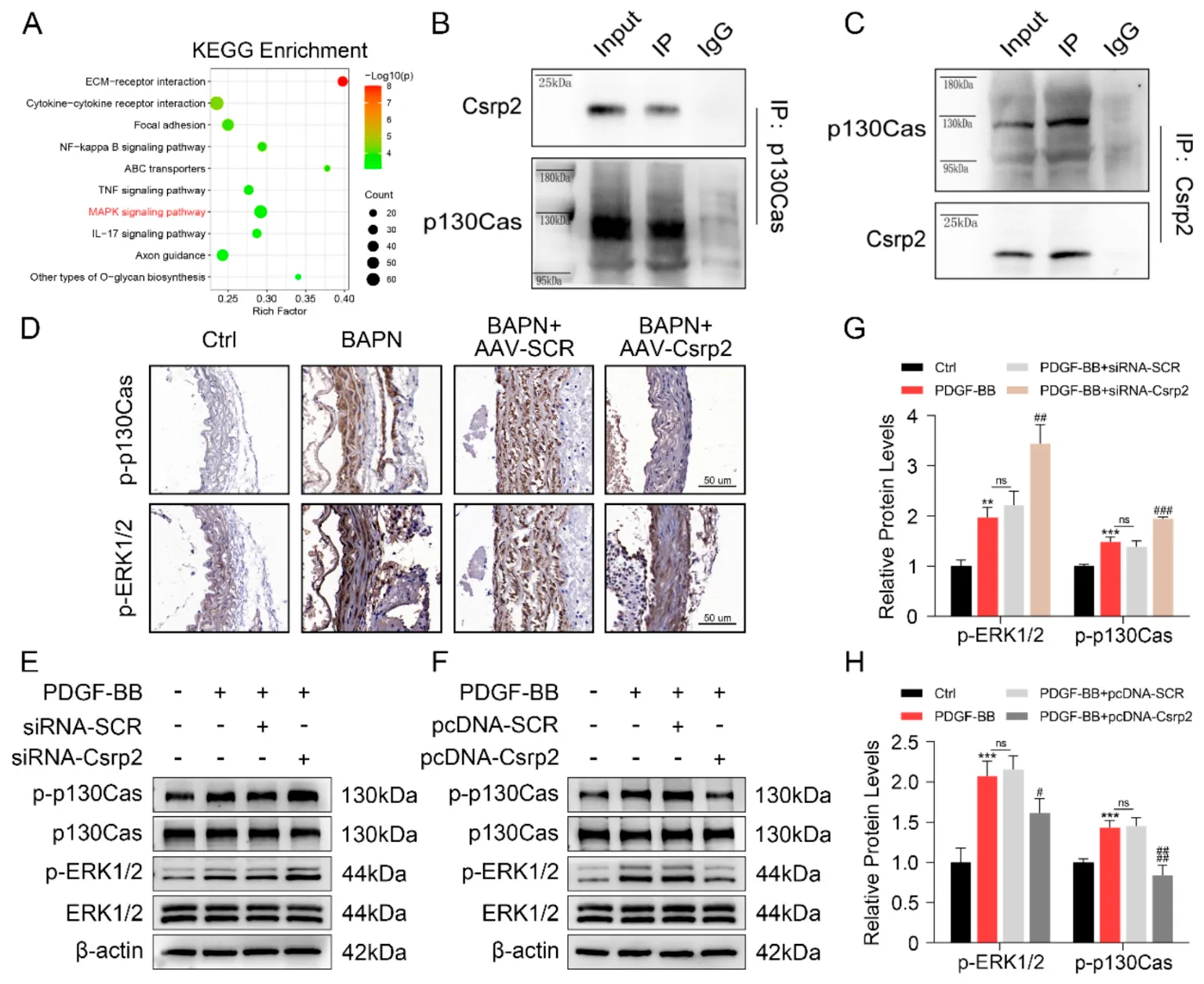
CSRP2 modulates p130Cas phosphorylation and ERK signaling pathway in VSMCs. (**A**) KEGG pathway enrichment bubble chart showing pathways enriched in mouse thoracic aortic tissues with CSRP2 overexpression. (**B**,**C**) Co-immunoprecipitation assay was performed to confirm the interaction between CSRP2 and p130Cas in VSMCs. (**D**) Immunohistochemistry was performed to detect p-p130Cas and p-ERK1/2 expression in mouse thoracic aortic tissues with indicated treatments. Scale bar = 50 μm. (**E**–**H**) CSRP2 modulates phosphorylation of p130Cas and ERK1/2 in VSMCs. MOVAS cells transfected with siRNA-CSRP2 or pcDNA-CSRP2 vectors as well as their corresponding scrambled controls were subjected to serum starvation for 24 h. Serum-starved cells with or without transfection were incubated with vehicle control or 10 ng/mL PDGF-BB for additional 24 h, followed by Western blot analysis and quantification. Original western blot images see supplementary Figure S4. Data are shown as mean ± standard deviation, ** *p* < 0.01, *** *p* < 0.001, versus control group, ^#^ *p* < 0.05, ^##^ *p* < 0.01, ^###^ *p* < 0.001, ^####^ *p* < 0.0001 versus PDGF-BB + empty vector group, ‘ns’ indicates non-significant difference, *n* = 3–5.

We noticed that the mitogen-activated protein kinase (MAPK) signaling pathway was one of the enriched pathways that was upregulated in human AD (Supplementary Material online, Figure S1A). Importantly, Kyoto encyclopedia of genes and genomes (KEGG) analysis of RNA sequencing data from mouse aortic tissues revealed that the MAPK signaling pathway was significantly enriched in tissues with CSRP2 overexpression (Figure 5A), indicating CSRP2’s potential role in modulating this pathway during AD formation. Additionally, the ERK1/2 signaling pathway is known to influence VSMC phenotypic switching [20]. Moreover, a previous report indicates an interaction between CSRP2 and p130Cas in colorectal cancer cells [21], and evidence also suggests that abnormal activation of p130Cas in various cancers promotes cell proliferation and migration [22]. We therefore investigated the interaction between CSRP2 and p130Cas in vascular smooth muscle cells (VSMCs) and assessed the impact of CSRP2 on the activation of p130Cas and ERK1/2.

Indeed, reciprocal Co-IP assays using antibodies against p130Cas (Figure 5B) and CSRP2 (Figure 5C) confirmed the direct interaction between CSRP2 and p130Cas. P130Cas generally exerts its function via phosphorylation. An antibody targeting the phosphorylated Tyr410 site was utilized to evaluate the phosphorylation status of p130Cas. IHC analysis using this antibody showed increased levels of p130Cas and ERK1/2 phosphorylation in aortic tissues treated with BAPN, which were significantly inhibited by CSRP2 overexpression (Figure 5D; Supplementary Material online, Figure S3A,B), indicating a regulator role of CSRP2 in p130Cas phosphorylation and ERK1/2 activation in the context of BAPN-induced AD formation. Moreover, Western blot analysis showed that phosphorylation levels of p130Cas and ERK1/2 were dramatically elevated in VSMCs by PDGF-BB (Figure 5E–H), which were further enhanced by the silencing of CSRP2 (Figure 5E and Figure 5G) but abolished by CSRP2 overexpression (Figure 5F and Figure 5H), respectively. The data indicate that CSRP2 directly interacts with p130Cas, regulating the phosphorylation of p130Cas and ERK1/2 during PDGF-BB-induced VSMC phenotypic switching and BAPN-induced AD formation.

### 3.5. CSRP2 Regulates VSMC Proliferation, Migration and Phenotypic Switching via p130Cas-Modulated ERK Signaling Pathway

**Figure 6:**
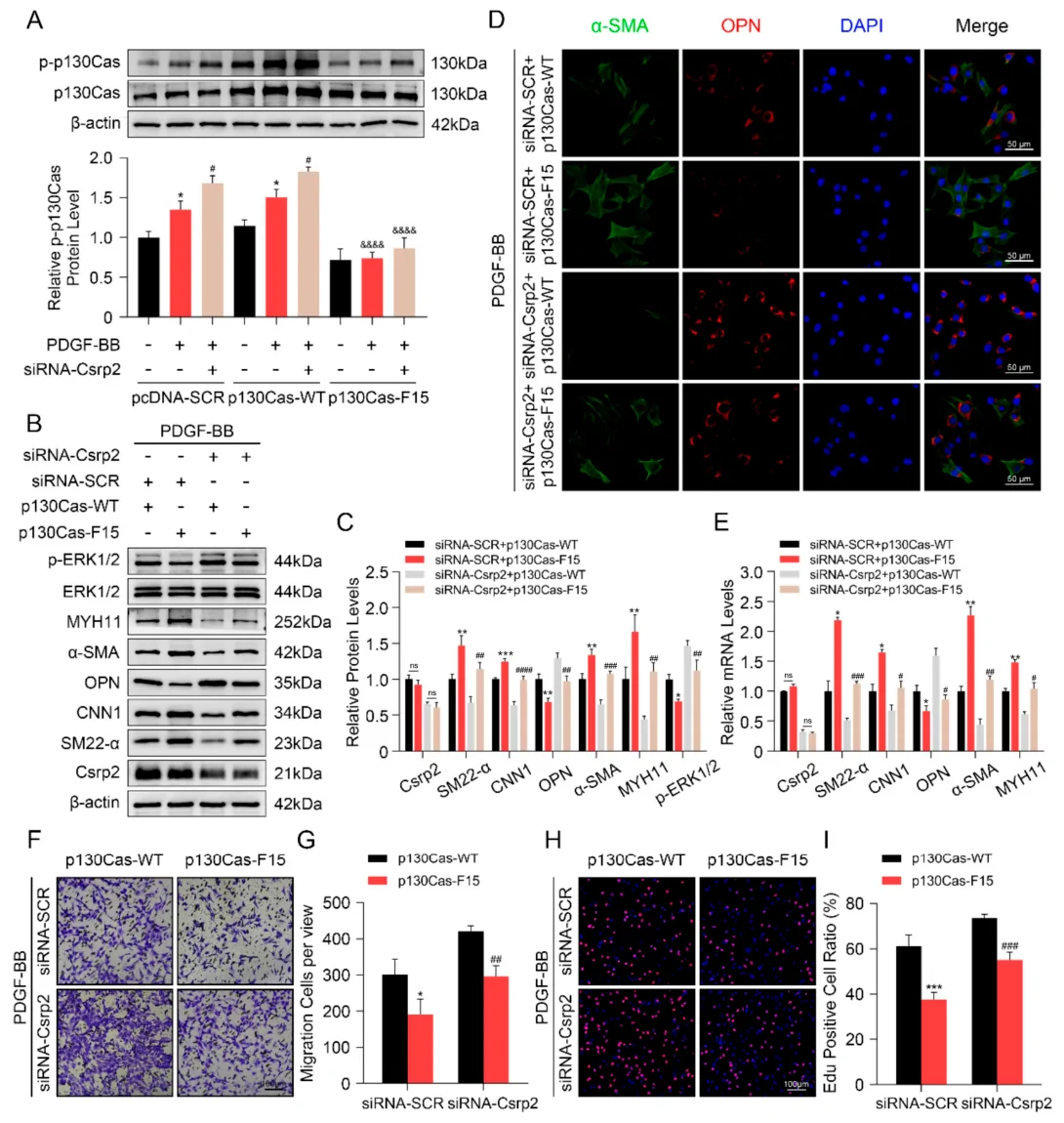
CSRP2 modulates ERK signaling pathways primarily through p130cas mediation to regulate VSMC proliferation, migration and phenotypic switch. MOVAS cells were co-transfected with siRNA-Csrp2, scrambled control, wild-type (pcDNA-p130Cas-WT) or mutated p130Cas overexpression (pcDNA-p130Cas-F15, phospho-dead mutation) pcDNA vectors as indicated. Transfected cells were subjected to serum starvation for 24 h, then incubated with vehicle control or 10 ng/mL PDGF-BB for additional 24 h, followed by different analyses. (**A**) Western blot analysis and quantification of total p130Cas and p-p130Cas in MOVAS cells with respective treatments, * *p* < 0.05 versus Ctrl group (black bars), ^#^ *p* < 0.05 versus PDGF-BB group (red bars), ^&&&&^ *p* < 0.0001 versus p130Cas-WT. Original western blot images see supplementary Figure S4. (**B**–**I**) p130Cas phosphorylation plays an important role in CSRP2-mediated VSMC phenotypic switching. MOVAS cells were co-transfected with siRNA-CSRP2, siRNA-SCR, pcDNA-p130Cas-WT or pcDNA-p130Cas-F15 as indicated. Transfected cells were subjected to serum starvation for 24 h, then incubated with 10 ng/mL PDGF-BB for additional 24 h. Cells with indicated treatments were subjected to Western blot analysis and quantification (**B**,**C**), immunofluorescence (**D**), RT-qPCR (**E**), transwell migration (**F**,**G**), and EdU staining (**H**,**I**) assays, respectively. Scale bar = 50 μm (D) and 100 μm (**F**,**H**). Data are shown as mean ± standard deviation, * *p* < 0.05, ** *p* < 0.01, *** *p* < 0.001 versus siRNA-SCR + p130Cas-WT group, ^#^ *p* < 0.05, ^##^ *p* < 0.01, ^###^ *p* < 0.001, ^####^ *p* < 0.0001 versus siRNA-CSRP2 + p130Cas-WT group, ‘ns’ indicates non-significant difference, *n* = 3.

Recent studies indicate that the phosphorylation of p130Cas activates ERK1/2, thereby promoting proliferation, migration, and tumor progression [23,24]. We hypothesized that the modulation of p130Cas would influence ERK signaling in the context of VSMC phenotypic transition. To validate this mechanism, scramble control (pcDNA-SCR), wild-type (p130Cas-WT), or phospho-dead p130Cas (p130Cas-F15) constructs were co-transfected into MOVAS cells with scramble siRNA (siRNA-SCR) or siRNA targeting CSRP2 (siRNA-CSRP2). We observed that while PDGF-BB treatment significantly increased p130Cas phosphorylation in MOVAS cells transfected with both pcDNA-SCR and p130Cas-WT vectors, which was further enhanced by CSRP2 knockdown, such regulatory effects disappeared in MOVAS cells transfected with p130Cas-F15 (Figure 6A). Moreover, while expression levels of p-ERK1/2 and VSMC synthetic marker OPN were significantly decreased by p130Cas-F15, the promotive effect of CSRP2 knockdown on ERK1/2 phosphorylation and OPN expression were blunted in MOVAS cells transfected with p130Cas-F15 (Figure 6B,C). Conversely, expression levels of VSMC contractile markers SM22-α, CNN1, α-SMA, and MYH11 were dramatically upregulated by p130Cas-F15; the inhibitory effect of CSRP2 inhibition on these marker protein expressions were abrogated in MOVAS cells transfected with p130Cas-F15 (Figure 6B,C). Furthermore, similar regulatory effects on these marker proteins were further confirmed using IF staining (Figure 6D; Supplementary Material online, Figure S3C,D) and RT-qPCR (Figure 6E), respectively. Finally, data from transwell assay (Figure 6F,G) and EdU staining (Figure 6H,I) showed that MOVAS cell migration and proliferation were significantly decreased by p130Cas-F15, and the promotive effect of CSRP2 knockdown on cell migration and proliferation was counterbalanced by overexpression of p130Cas-F15. The above data have collectively shown that p130Cas phosphorylation is critical for CSRP2-mediated VSMC phenotypic switching.

**Figure 7:**
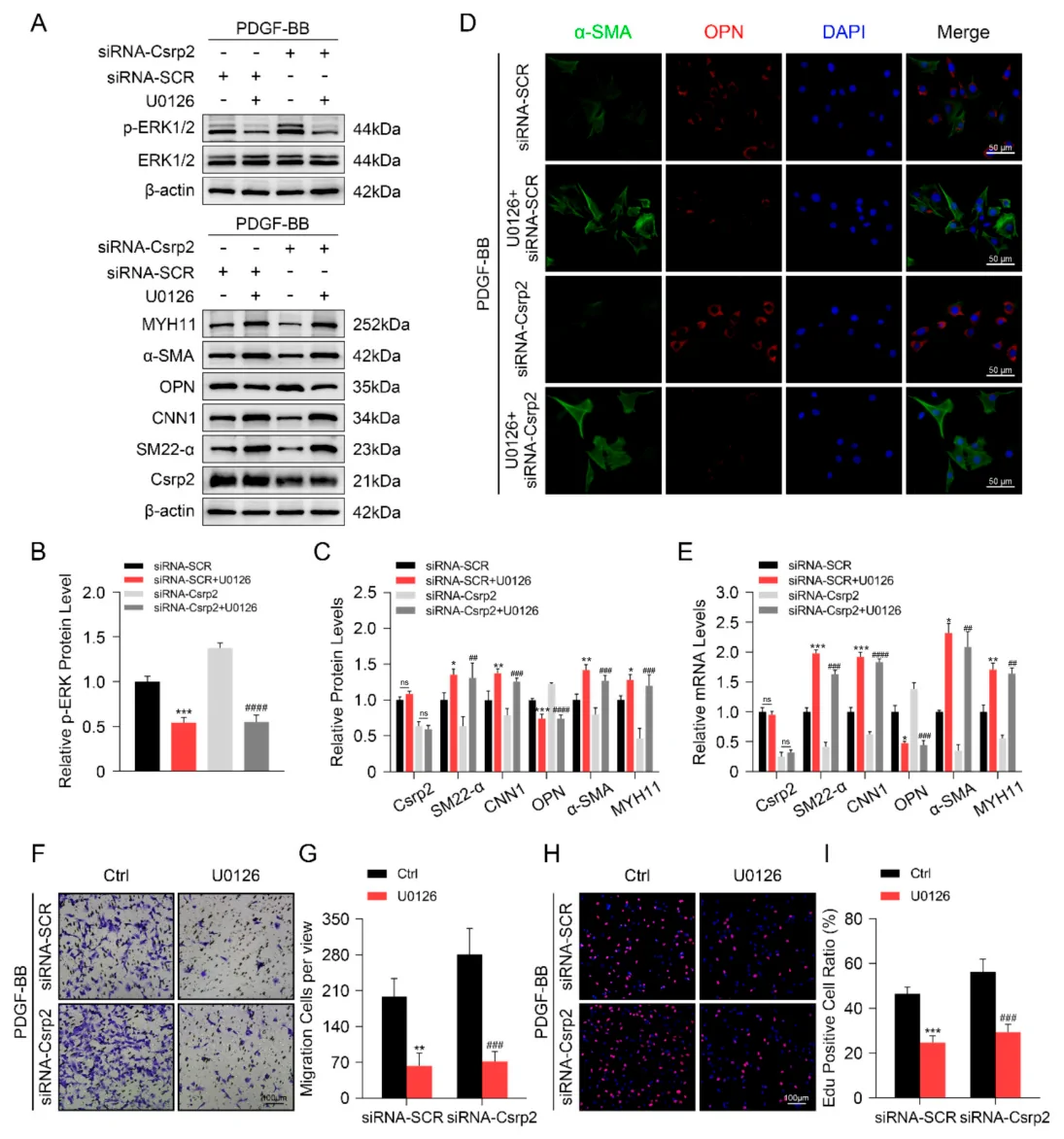
CSRP2 inhibition regulates VSMC phenotypic switching primarily via activating ERK signaling pathways. MOVAS cells transfected with siRNA-CSRP2 or scrambled control were subjected to serum starvation for 24 h. Serum-starved cells were incubated with vehicle control or 10 µM U0126 (a potent and selective inhibitor of MAPK kinases) in the presence of 10 ng/mL PDGF-BB for additional 24 h, followed by different analysis. Cells with indicated treatments were subjected to Western blot analysis and quantification (**A**–**C**), original western blot images see supplementary Figure S4, immunofluorescence (**D**), RT-qPCR (**E**), transwell migration (**F**,**G**), and EdU staining (**H**,**I**) assays, respectively. Scale bar = 50 μm (**D**) and 100 μm (**F**,**H**). Data are shown as mean ± standard deviation, * *p* < 0.05, ** *p* < 0.01, *** *p* < 0.001 versus siRNA-SCR group, ^##^ *p* < 0.01, ^###^ *p* < 0.001, ^####^ *p* < 0.0001 versus siRNA-CSRP2, ‘ns’ indicates non-significant difference, *n* = 3.

To further explore a potential role of ERK1/2 signaling pathway in CSRP2-mediated VSMC phenotypic switching, U0126, a potent and selective inhibitor of MAPK kinases such as MEK1/2 and ERK1/2, was used to treat MOVAS cells transfected with siRNA-SCR or siRNA-CSRP2. Indeed, whereas U0126 markedly reduced the level of p-ERK1/2 in MOVAS cells, it had no significant impact on CSRP2 expression (Figure 7A–C). Consequently, while VSMC contractile markers SM22-α, CNN1, α-SMA, and MYH11 were significantly increased by U0126, the inhibitory effects of CSRP2 knockdown on these proteins were abolished by U0126 (Figure 7A,C,E). Meanwhile, we observed an opposite regulatory effect of U0126 on the synthetic marker OPN (Figure 7A,C,E). Such observations were further confirmed by IF staining using antibodies against α-SMA and OPN (Figure 7D; Supplementary Material online, Figure S3E,F). Functionally, data from the transwell assay (Figure 7F,G) and EdU staining (Figure 7H,I) indicate that U0126 significantly inhibited MOVAS cell migration and proliferation. Furthermore, the regulatory effects of CSRP2 knockdown on these processes were negated in the presence of U0126. These results suggest that activation of the ERK1/2 signaling pathway is required for CSRP2-mediated VSMC phenotypic switching.

## 4. Discussion

CSRP2, part of the CRP family, is characterized by two functional LIM domains linked to amino acid-rich repeats, crucial for its biological functions. CSRP2 is predominantly expressed in VSMCs and has been identified as one of the most significant DEGs within VSMC clusters of aneurysmal infrarenal abdominal aorta [14]. Moreover, Wei et al. reported a protective role for CSRP2 in injury-induced neointimal hyperplasia [13]. Our study indicates for the first time that CSRP2 is a novel protector against the development and rupture of AD. Specifically, CSRP2 is markedly downregulated in both human and murine AD, and CSRP2 overexpression reduces AD incidence and protects AD from rupture. Mechanistically, we show that CSRP2 counterbalances PDGF-BB- or BAPN-induced VSMC phenotypic switching. Further mechanistic studies showed that CSRP2 could directly interact with p130Cas and regulate its phosphorylation, which in turn modulates ERK signaling pathways and prevents VSMC phenotypic transition from contractile to synthetic phenotype, thereby attenuating AD progression and protecting AD from rupture. While systemic factors such as hypertension and genetic predispositions are the primary initiators of AD, our data position CSRP2 as a pivotal endogenous protective factor. Its downregulation appears to be a key event that accelerates disease progression by exacerbating VSMC dysfunction within the context of ongoing pathological insults. Our findings comprehensively suggest that activation or overexpression of CSRP2 could be a novel target for early intervention for AD patients.

CSRP2 may exert context-dependent roles in distinct aortic pathologies. While our study identifies CSRP2 as a protective factor against AD, Chen et al. reported that CSRP2 deficiency attenuates Ang II-induced abdominal aortic aneurysm (AAA) formation [25]. This apparent contradiction likely stems from fundamental differences in disease mechanisms: AD is driven by acute medial degeneration, characterized by extensive elastic fiber fragmentation and loss of VSMC contractility. In contrast, AAA involves chronic expansive remodeling driven by persistent inflammation, proteolysis, and excessive ECM deposition. Furthermore, Chakraborty et al. demonstrated that despite both being AD models, BAPN and Ang II evoke distinct temporal activation of the STING-IRF3-EZH2 axis in VSMC [26], emphasizing that model-specific signaling and disease timelines critically shape CSRP2’s net effect. Thus, CSRP2 is protective when medial integrity is threatened (AD), yet potentially maladaptive when pathological ECM turnover predominates (AAA). These considerations highlight the complexity of CSRP2’s role in aortic diseases and underscore the need for targeted therapeutic strategies based on specific pathological contexts.

CRPs are involved in the regulation of the cytoskeleton through interactions with several proteins, including the actin cross-linking protein α-actin, the adhesion plaque protein zyxin, and the scaffold protein p130Cas [27,28]. Co-IP and ELISA assays confirmed a direct association between CSRP2 and F-actin [29]. Moreover, CSRP2 has the capacity to sequester p130Cas from focal adhesions, thereby regulating lamellipodia formation and diminishing the motility of VSMCs [27]. These observations support a role for CSRP2 in cytoskeletal regulation and cell motility, two critical processes linked to the phenotypic switching in VSMCs.

It has been suggested that VSMC phenotypic transformation is one of the essential mechanisms underpinning the pathological processes of AD. The typical aortic media is characterized by a densely organized structure of VSMCs and an extracellular matrix that is rich in elastic fibers. In patients with AD, a notable increase in the number of synthetic VSMCs was noted, contributing to reduced aortic elasticity and heightened risk of aortic rupture [30]. Recent research has provided direct evidence to support the functional roles of VSMC contractile genes in AD and aortic aneurysm. Specifically, a previous study showed that SM22-α can inhibit VSMC phenotypic switching, thus preventing AA progression [31]. In the current study, a variety of gain- and loss-of-function experiments were performed to investigate the biological role of CSRP2 in AD both in vitro and in vivo. Our data showed that SM22-α, along with other VSMC contractile marker proteins, including CNN1, α-SMA, and MYH11, were positively regulated by CSRP2 in the context of AD. Meanwhile, we also found that VSMC synthetic marker OPN was negatively modulated by CSRP2 in the same experimental settings. These data suggest that CSRP2 is a novel regulator in governing VSMC phenotypic switching in the context of AD.

Further investigations were performed to elucidate the molecular mechanisms through which CSRP2 modulates VSMC phenotypic switching. P130Cas is a scaffold protein with various structural motifs, such as a proline-rich domain, an SH3 domain, a substrate domain with 15 YxxP repeats, and a C-terminal domain related to the Cas family. The main post-translational modifications of p130Cas involve phosphorylation at tyrosine and serine/threonine residues, notably at the tyrosine kinase sites Tyr165, Tyr249, and Tyr410 [21]. Phosphorylation of p130Cas is crucial for regulating cytoskeletal remodeling, enhancing cell migration, and contributing significantly to vascular disease pathogenesis [27,32]. Intracellular signaling pathways, including ERK, a classical MAPK signal transduction pathway, are essential for cell migration and proliferation in mammalian cells. Recent studies indicate that CSRP2 mediates the Ang-II-activated ERK1/2 signaling pathway, resulting in abnormal aortic ECM remodeling [25]. Consistent with these previous findings, we confirmed the direct interaction of CSRP2 with p130Cas, and an inhibitory effect of CSRP2 on the phosphorylation of p130Cas and the activation of the ERK1/2 pathway.

P130Cas is a recognized multifunctional scaffold protein in signaling networks, influencing various cellular processes [22]. P130Cas is crucial for regulating cell migration in various cell types, including carcinoma, glioma, and endothelial cells [33,34]. Neuropilin-1 facilitates PDGF-induced VSMC migration via p130Cas [35]. Ang-II promotes the association of Src with p130Cas, leading to the phosphorylation of p130Cas and enhanced migration of VSMCs. Nitric oxide, on the other hand, decreases cell motility through the enhancement of PTP-PEST activity and dephosphorylation of its substrate, p130Cas [36,37]. The functional significance of the p130Cas substrate domain has been demonstrated. In this study, overexpression of a phospho-dead p130Cas mutant could inhibit PDGF-BB-induced VSMC proliferation, migration and phenotypic switching. Similarly, zyxin has been shown to interact with the substrate domain of p130Cas, and fibroblasts lacking zyxin exhibit increased migration capabilities [38].

**Figure 8:**
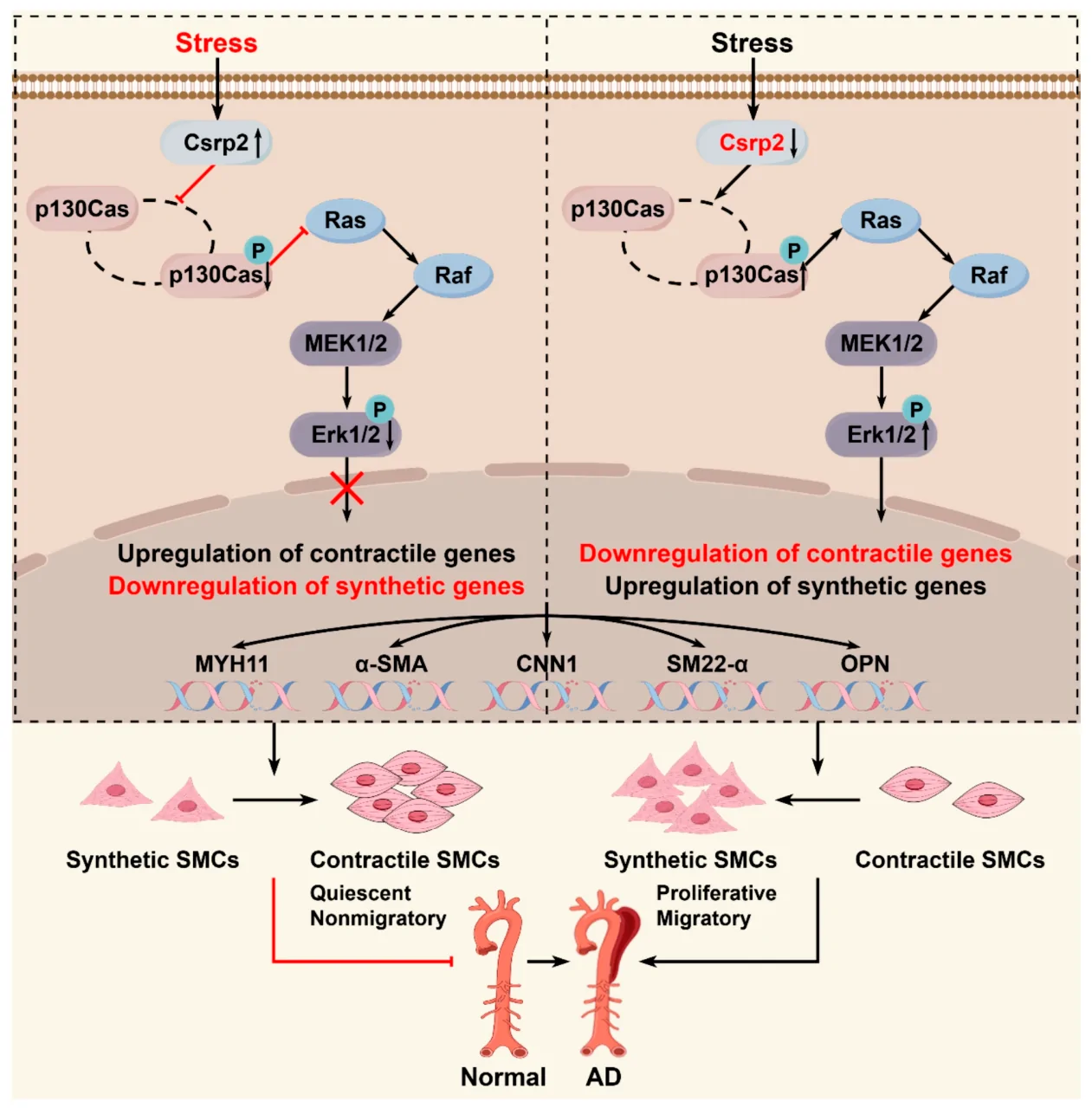
Schematic diagram showing the mechanism of action of CSRP2 in AD. Under physiological condition, CSRP2 interacts with p130Cas and inhibits the phosphorylation of p130cas, which in turn suppresses the ERK signal pathway, thereby preserving VSMC contractile phenotype. Conversely, CSRP2 expression is downregulated under pathological conditions, and decreased CSRP2 promotes p130Cas phosphorylation and activates ERK signal pathways, which in turn enhances VSMC switching into a synthetic phenotype, thereby promoting AD formation. Created by Figdraw.com.

Multiple studies have indicated that the ERK1/2 signaling pathway is linked to VSMC phenotypic switching and plays a role in VSMC dysfunction, vasoconstriction, and vascular remodeling. Activation of ERK1/2 was significantly elevated in human AA lesions and in aortic tissues from Ang II-infused mice with AA. In contrast, substances that inhibited AA formation effectively prevented ERK1/2 activation [39,40,41]. Inhibition of ERK1/2 by U0126 resulted in decreased expression of Col III and matrix metalloproteinase-2, as well as reduced matrix metalloproteinase-2 activity in VSMCs [25]. Indeed, we indicated a correlation between the VSMC phenotypic switching and the ERK1/2 signaling pathway. Our study shows that U0126-mediated ERK1/2 inhibition enhances VSMC contractile gene expression and reduces VSMC synthetic genes. Similar effects were observed using phosph-dead p130Cas mutant. Taken together, these findings indicate that CSRP2 regulates VSMC proliferation, migration and phenotypic switching primarily via p130Cas-modulated ERK signaling pathways. The procedure is schematically depicted in Figure 8.

We acknowledge several limitations of this study. First, although our clinical findings were corroborated by independent public datasets, the modest size of our primary cohort warrants validation in large-scale, multi-center studies. Second, the supraphysiological CSRP2 overexpression was employed to establish the sufficiency of the CSRP2/p130Cas/ERK axis in conferring protection. Further dose-titration studies are required to define the minimal, physiologically relevant protective threshold, thereby aligning more closely with potential clinical translation. Third, while our data implicate ERK signaling as the primary mediator of CSRP2’s protective effect, we did not experimentally exclude the contribution of other p130Cas downstream effectors or parallel MAPK pathways. Future studies employing pathway-specific inhibitors or genetic interference are required to fully delineate the role of these alternative cascades. Fourth, although MOVAS immortalized cell lines retain core features of the contractile phenotype, they may not fully mirror the phenotypic plasticity and complex behaviors of primary VSMC isolated from aortic tissue. Current cellular findings require further validation in primary VSMC cultures to confirm their physiological relevance in the context of AD. Finally, the lack of in vitro converse rescue experiment and in vivo loss-of-function validation. Our two orthogonal complementary approaches (Figure 6 and Figure 7) provide robust genetic and pharmacological evidence, thereby substantially mitigating the absence of a converse rescue. While CSRP2 overexpression is sufficient to attenuate AD development, we cannot claim necessity without genetic loss-of-function models. Addressing this via CSRP2 knockout or deficient mouse is beyond the current scope but will be a future priority.

## 5. Conclusions

The present study identified CSRP2 as a significant regulator and a promising target for early intervention in AD pathogenesis. We show that CSRP2 is linked to a reduced risk of AD, and it regulates VSMC phenotypic switching both in vitro and in vivo in the context of AD formation. Mechanistic studies suggest that CSRP2 modulates p130Cas phosphorylation and ERK activation, thereby regulating VSMC proliferation, migration, and phenotypic switching. We also indicate the translational potential of targeting CSRP2 and identify a novel mechanism involving the CSRP2/p130Cas/ERK axis underlying AD formation, therefore offering a novel target for early intervention for AD patients by specifically targeting this signal axis.

## Figures and Tables

**Table 1:** Oligonucleotide primer sets for RT-qPCR.

Name	Sequence (5′-3′)	Length
Human *CSRP2* F	TGGGAGGACCGTGTACCAC	19
Human *CSRP2* R	CCGTAGCCTTTTGGCCCATA	19
Murine *CSRP2* F	GCTGCGGAGAAGATCATTGG	20
Murine *CSRP2* R	GTTCTTTGCGTAGCACCCTT	20
Murine *TAGLN* F	TTAGCCTGCCTCACAAATGC	20
Murine *TAGLN* R	GGGCTGAGGCTAAGGATAGG	20
Murine *SPP1* F	CAGCCATGAGTCAAGTCAGC	20
Murine *SPP1* R	TGTGGCTGTGAAACTTGTGG	20
Murine *CNN1* F	CGCATCGGGAACAACTTCAT	20
Murine *CNN1* R	GGTGCCAGTTCTGAGTTGAC	20
Murine *ACTA2* F	GTCCCTCTATGCCTCTGGAC	20
Murine *ACTA2* R	AAGGAATAGCCACGCTCAGT	20
Murine *MYH11* F	AAGAGCTGGAGAGGACCAAC	20
Murine *MYH11* R	CATGCACGTTCTTGCCTACA	20
Human *GAPDH* F	GGAGCGAGATCCCTCCAAAAT	21
Human *GAPDH* R	GGCTGTTGTCATACTTCTCATGG	23
Murine *GAPDH* F	AGGTCGGTGTGAACGGATTTG	21
Murine *GAPDH* R	GGGGTCGTTGATGGCAACA	19

## Data Availability

The data used to support this study’s findings are available from the corresponding author upon reasonable request.

## Supplementary Materials

The following supporting information can be downloaded at https://www.mdpi.com/article/10.3390/biom16081101/s1, Figure S1. Pathway enrichment analysis (related to Figure 1); Figure S2. Immunostaining analysis (related to Figure 2); Figure S3. Quantitative analysis of the relative protein expression levels for immunostaining (related to Figure 5D, Figure 6D and Figure 7D); Figure S4. The original Western blot images.

## Abbreviations and Acronyms

AD	aortic dissection
VSMCs	vascular smooth muscle cells
CSRP2	cysteine-rich protein 2
BAPN	β-aminopropionitrile monofumarate
PDGF-BB	platelet-derived growth factor subunit BB
IHC	immunohistochemistry
IF	immunofluorescence
WB	western blotting
RT-qPCR	quantitative real-time PCR
H&E	hematoxylin and eosin
EVG	elastin Verhoeff-van-Giessen
Co-IP	co-immunoprecipitation
ECM	extracellular matrix
GEO	gene expression omnibus
GSEA	gene set enrichment analysis
SM22-α	smooth muscle protein 22-α
CNN1	calponin 1
α-SMA	α-smooth muscle actin
MYH11	myosin heavy chain 11
GO	gene ontology
OPN	osteopontin
KEGG	Kyoto encyclopedia of genes and genomes
MAPK	mitogen-activated protein kinase
ERK	extracellular signal-regulated kinases
