# Supplementary material for: The Role of Cysteine-Rich Protein 2 in Aortic Dissection: Implications for VSMC Phenotypic Modulation—CSRP2 Impedes the Progression of Aortic Dissection
Source: Biomolecules. 2026 Jul 28;16(8):1101. doi: 10.3390/biom16081101 (PMC13511068; doi:10.3390/biom16081101)
Supplement: Supplementary file 1 [file biomolecules-16-01101-s001.zip › biomolecules-4352668-Supplemental Figure S1-3.pdf]

Supplementary figures and figure legends

Figure S1-A

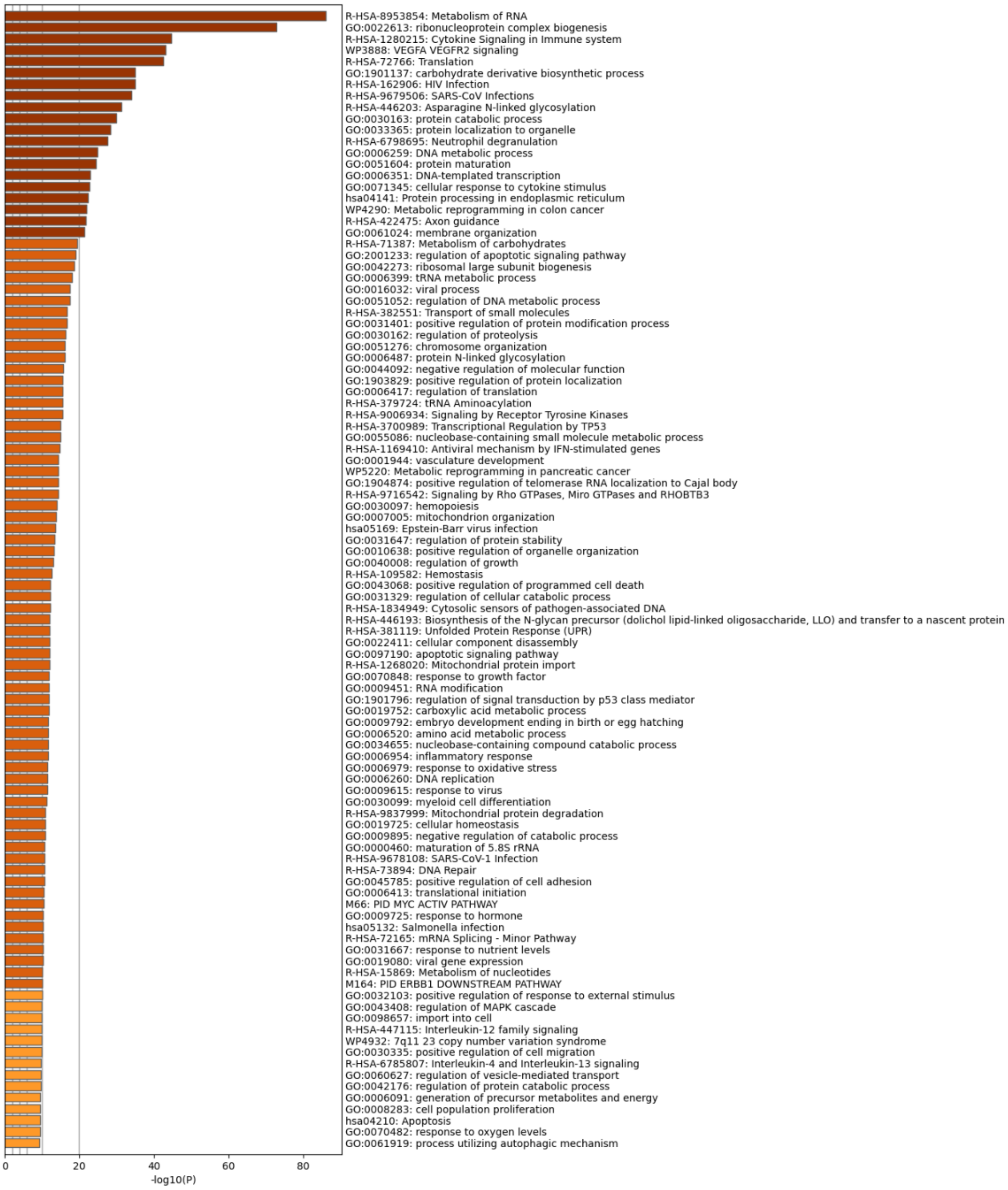

Figure S1-B

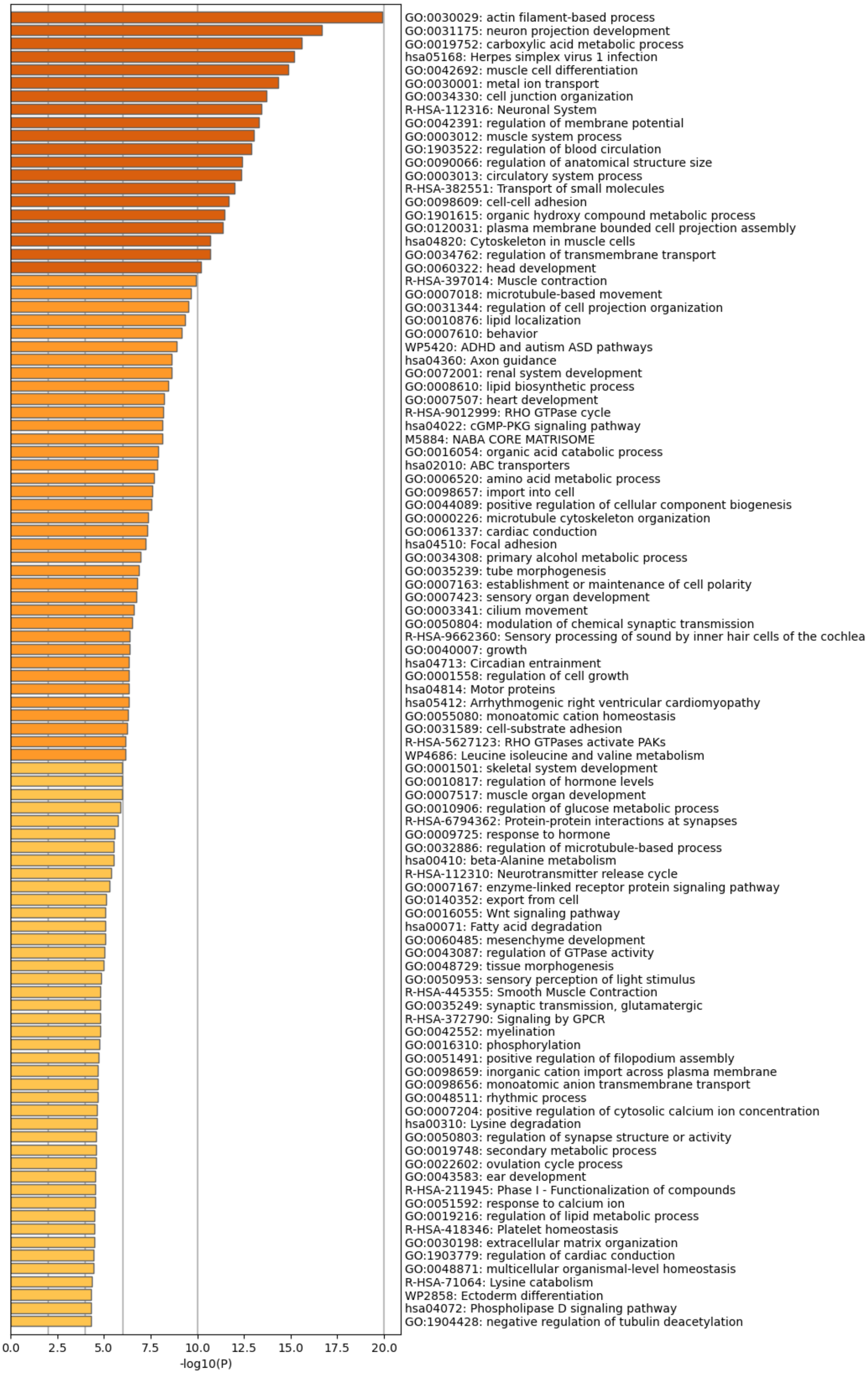

Figure S1. Pathway enrichment analysis (related to Figure 1).

Five pairs of normal healthy and human AD aortic samples were collected and subjected to RNA sequencing analysis. Upregulated (A) and downregulated (B) differently expressed genes in human AD were subjected to pathway enrichment analysis using free online software Metascape (https://metascape.org/gp/index.html#/main/step1). Top 100 significantly enriched pathways were presented here.

Figure S2

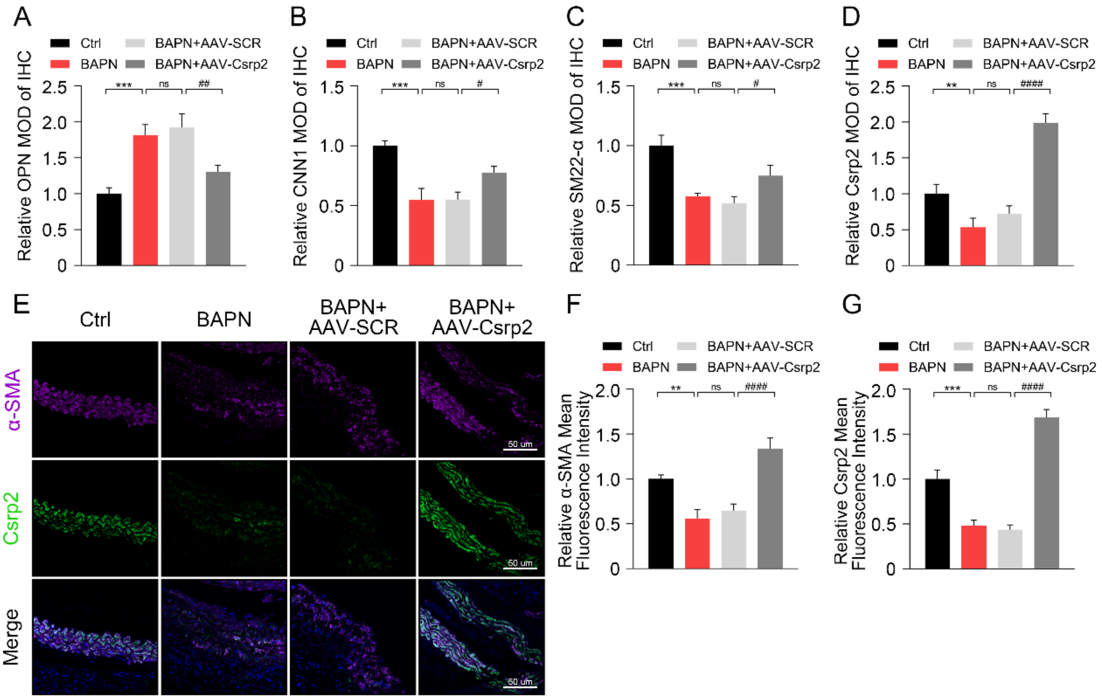

Figure S2. Immunostaining analysis (related to Figure 2).

(A-D) Quantitative analysis of the relative protein expression levels of osteopontin (OPN, A), calponin 1 (CNN1, B), smooth muscle 22-alpha (SM22-α, C), and CSRP2 (D) in mouse aortic tissues with indicated treatments. (E-G) Immunofluorescence (IF) staining showing CSRP2 and alpha-smooth muscle actin (α-SMA) in mouse aortic tissues with indicated treatments. Relative protein expression level was measured as mean optical density (MOD for IHC staining, A-D) or mean fluorescence intensity (for IF staining, F-G), respectively. Data are shown as mean ± standard deviation, $^{**}p < 0.01$,

***$p < 0.001$ versus Ctrl group, #$p < 0.05$, ##$p < 0.01$, ####$p < 0.0001$ versus BAPN+AAV-SCR group, 'ns' indicates non-significant difference, n = 5-9.

Figure S3

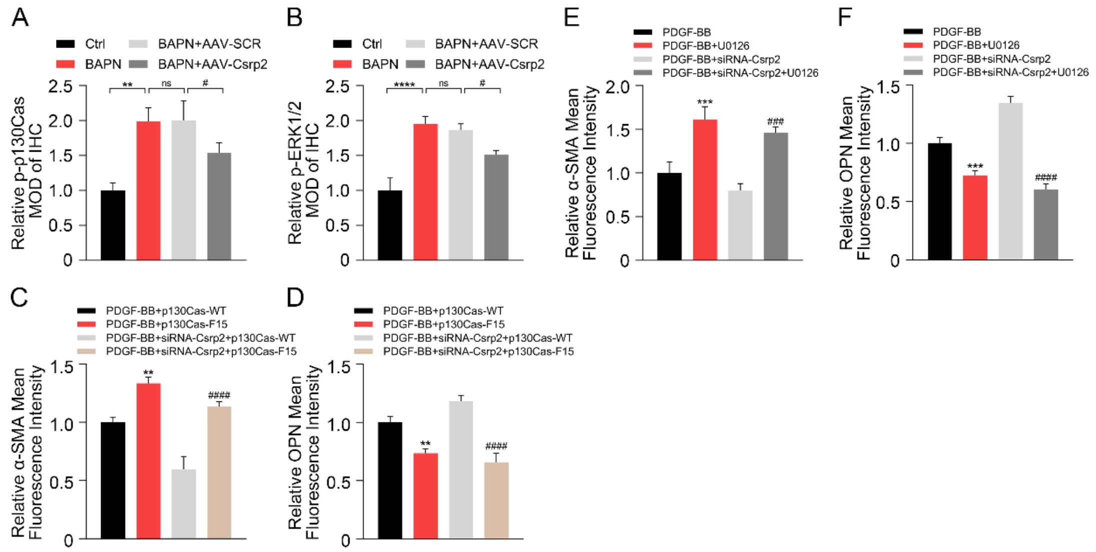

Figure S3. Quantitative analysis of the relative protein expression levels for immunostaining (related to Figure 5D, 6D and 7D).

(A-B) Quantitative analysis of the relative protein expression levels of p-p130Cas (A) and p-ERK1/2 (B) measured as mean optical density (MOD for IHC staining) (related to Figure 5D). (C-D) Quantitative analysis of the relative protein expression levels of alpha-smooth muscle actin (α-SMA, C) and osteopontin (OPN, D) measured as mean fluorescence intensity for immunofluorescence (IF) staining (related to Figure 6D). (E-F) Quantitative analysis of the relative protein expression levels of α-SMA (E) and OPN (F) measured as mean fluorescence intensity for immunofluorescence (IF) staining (related to Figure 7D).
