# Supplementary material for: The Role of Cysteine-Rich Protein 2 in Aortic Dissection: Implications for VSMC Phenotypic Modulation—CSRP2 Impedes the Progression of Aortic Dissection
Source: Biomolecules. 2026 Jul 28;16(8):1101. doi: 10.3390/biom16081101 (PMC13511068; doi:10.3390/biom16081101)
Supplement: Supplementary file 1 [file biomolecules-16-01101-s001.zip › biomolecules-4352668-Supplemental Figure S4.pdf]

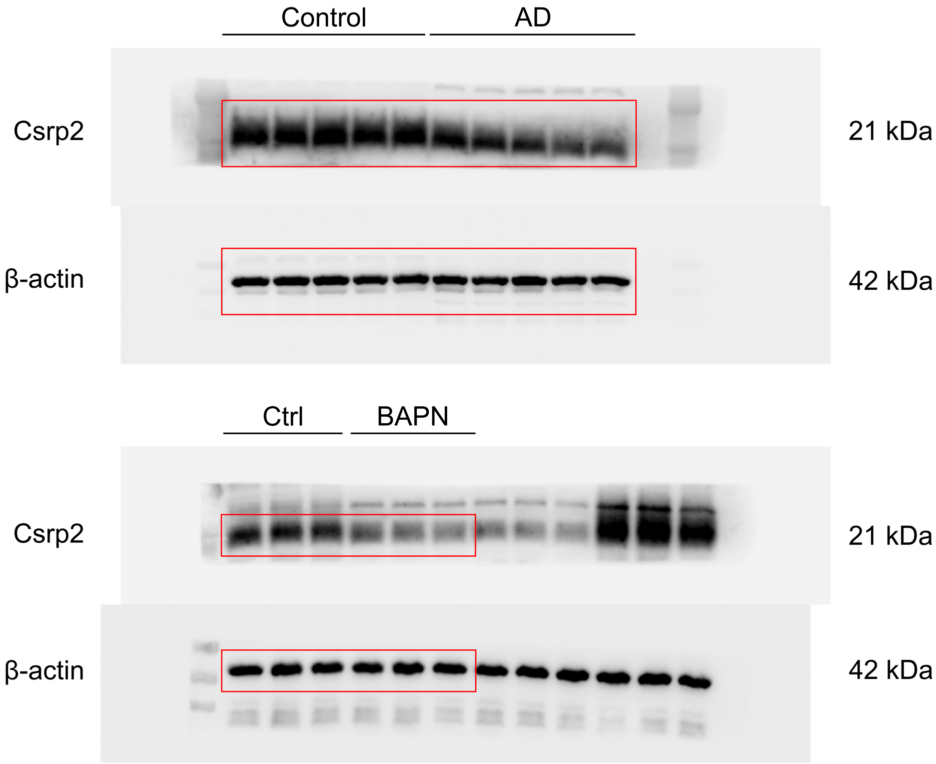

Raw blots in Figure 1.

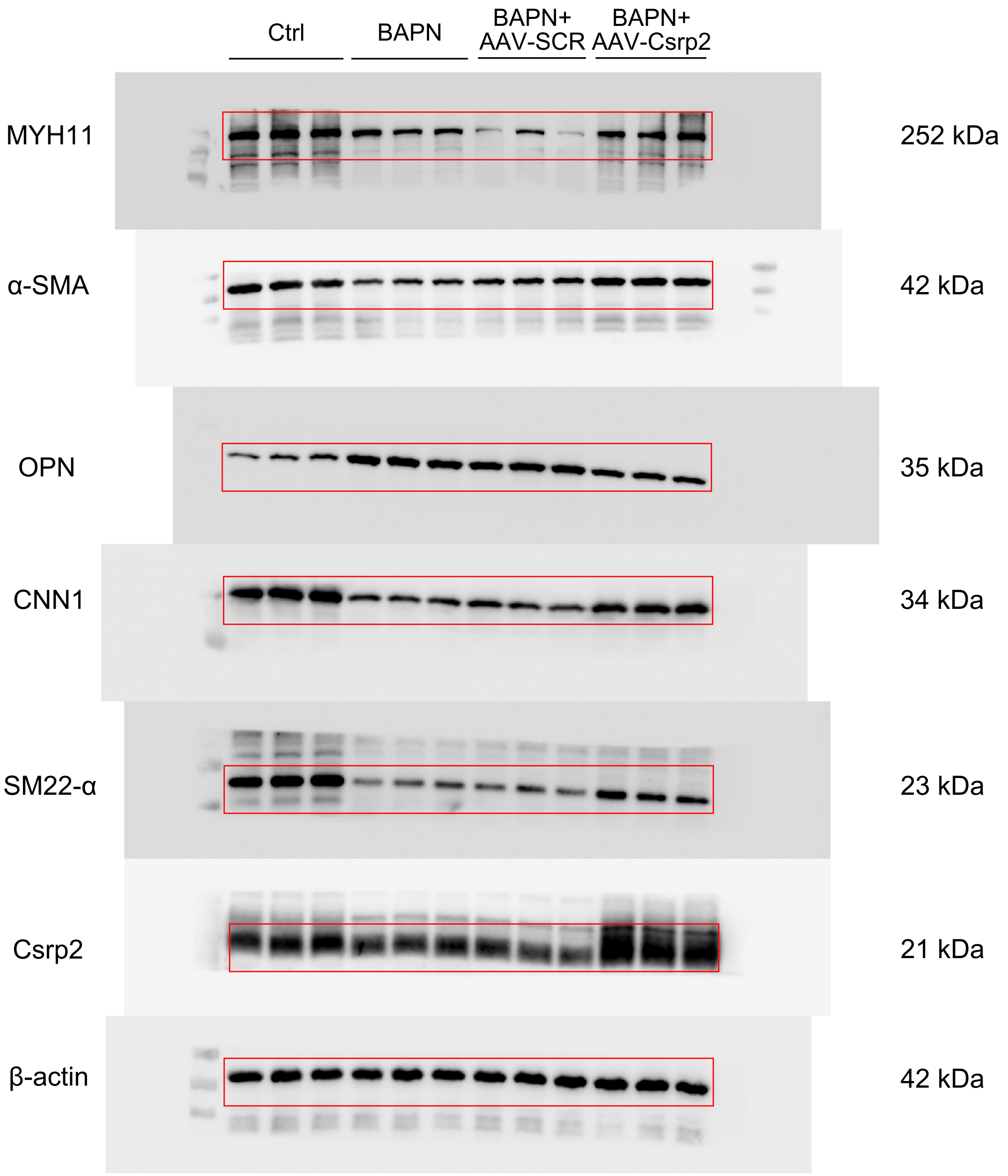

Raw blots in Figure 2.

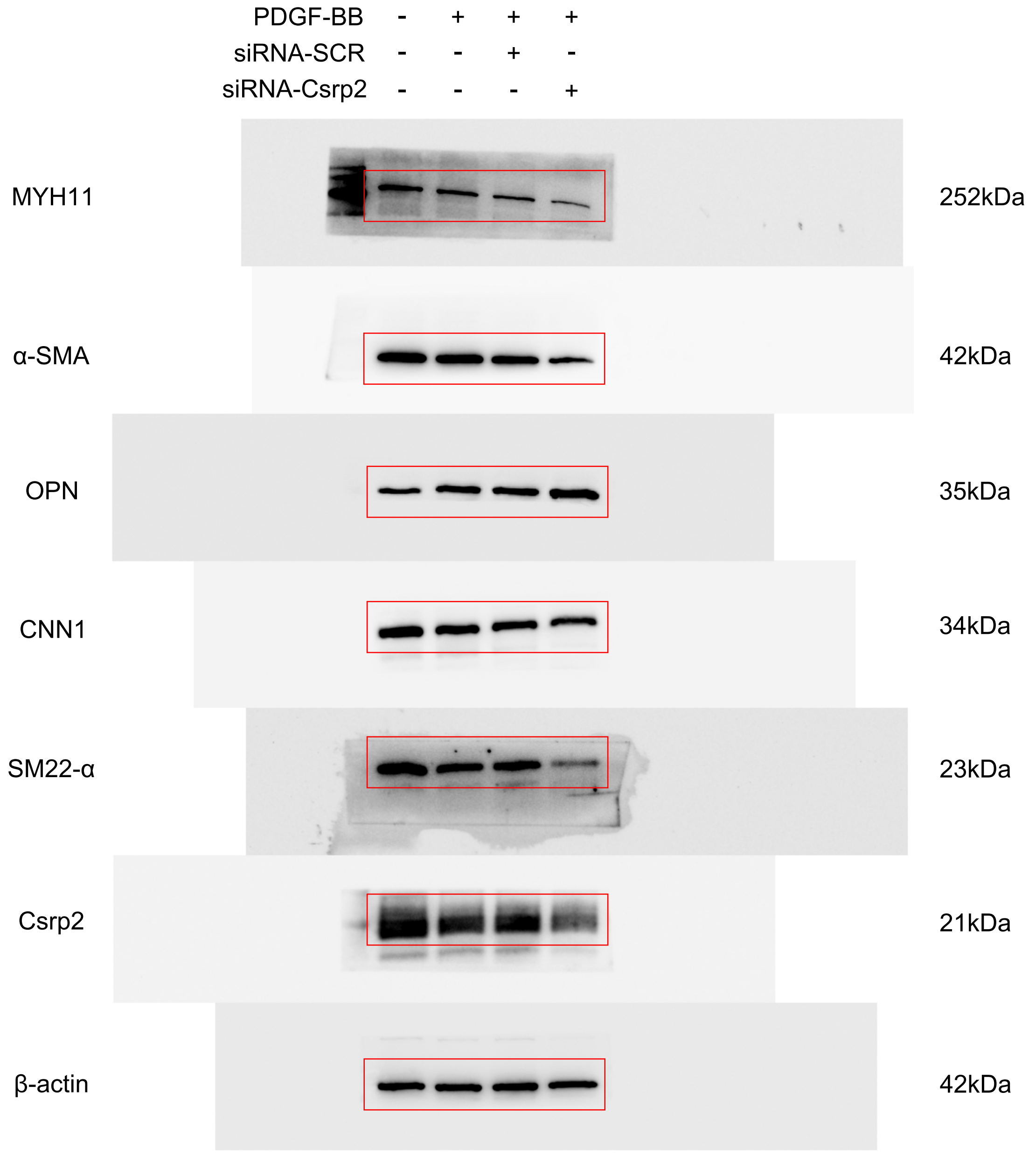

Raw blots in Figure 3.

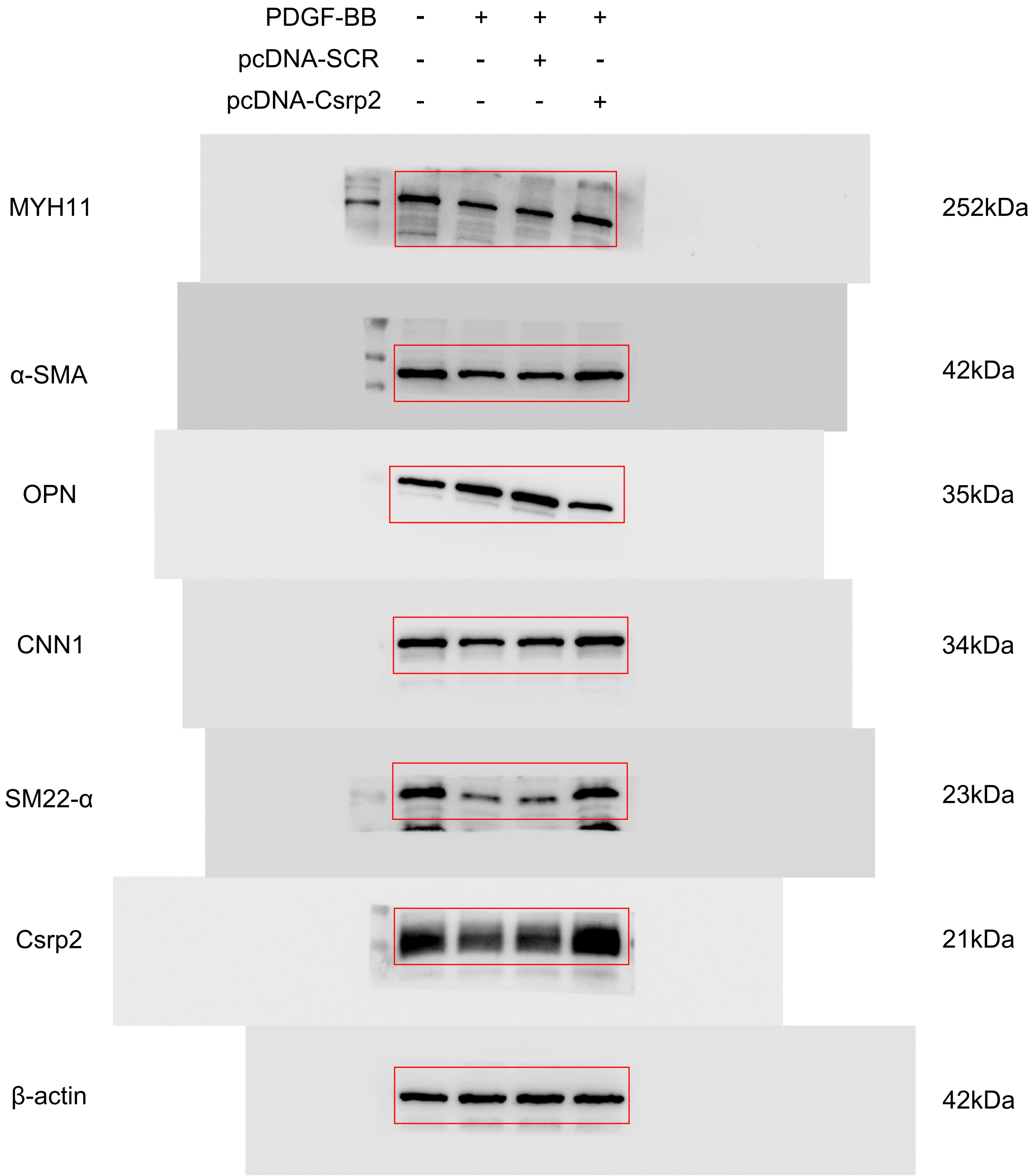

Raw blots in Figure 4.

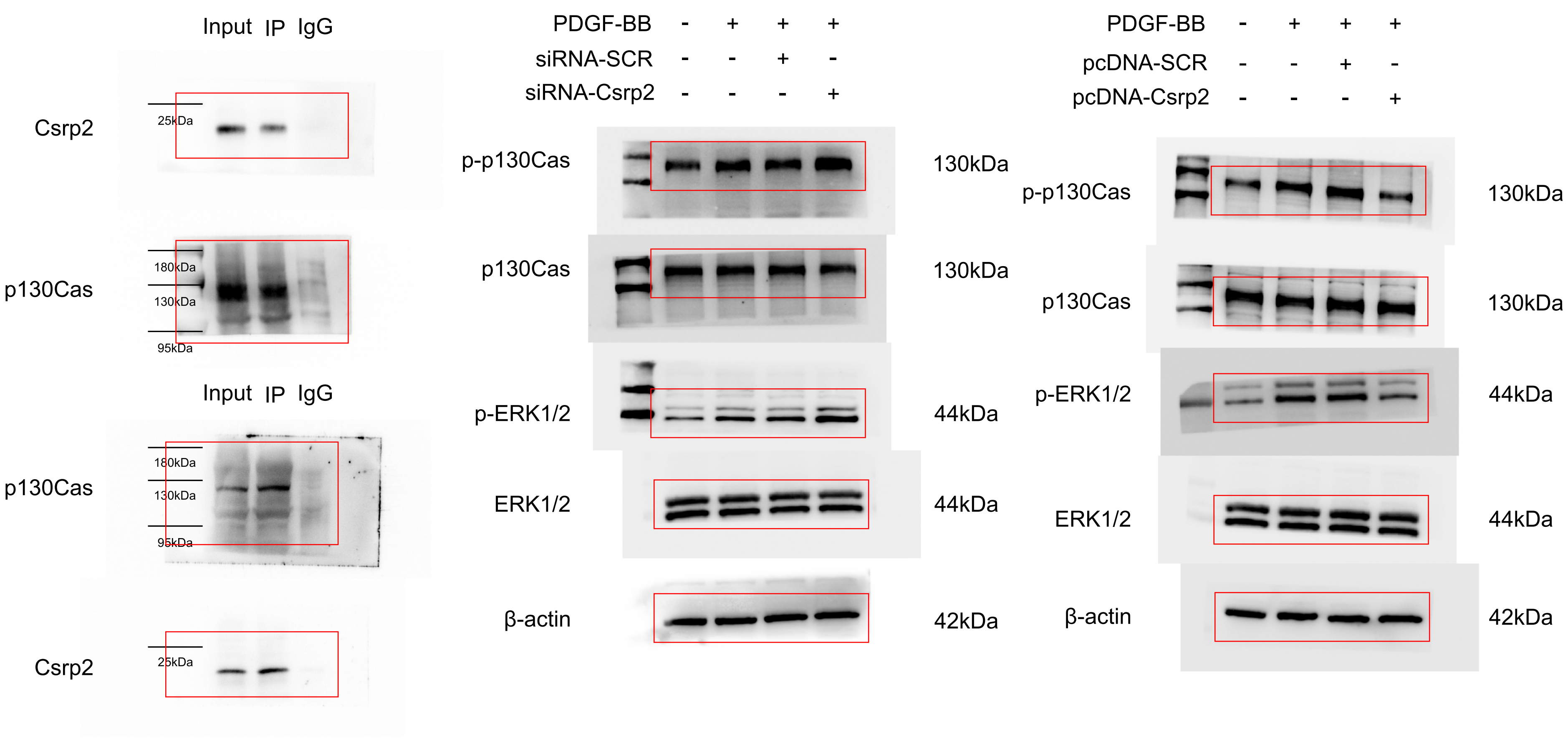

Raw blots in Figure 5.

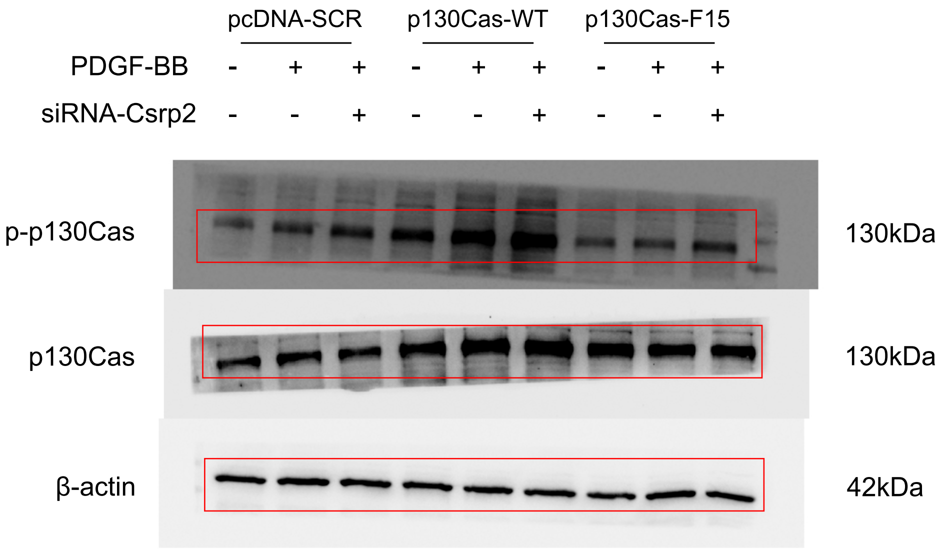

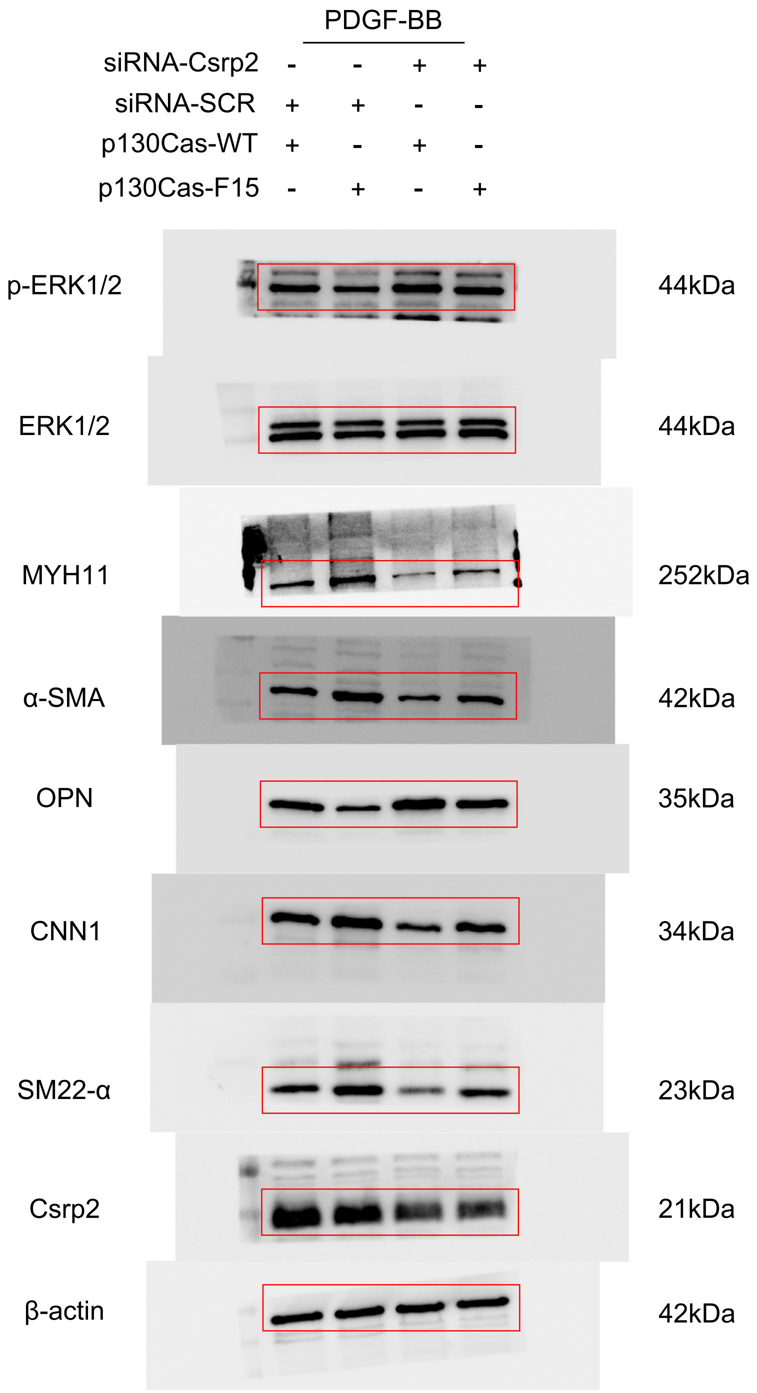

Raw blots in Figure 6.

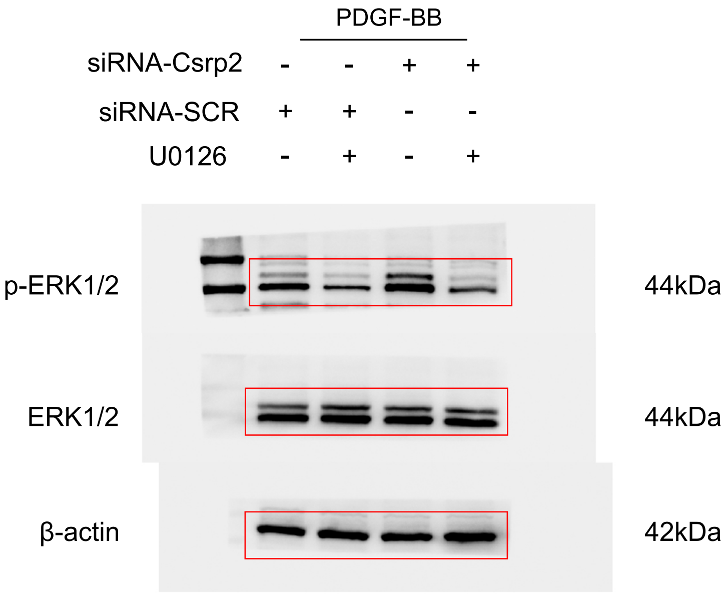

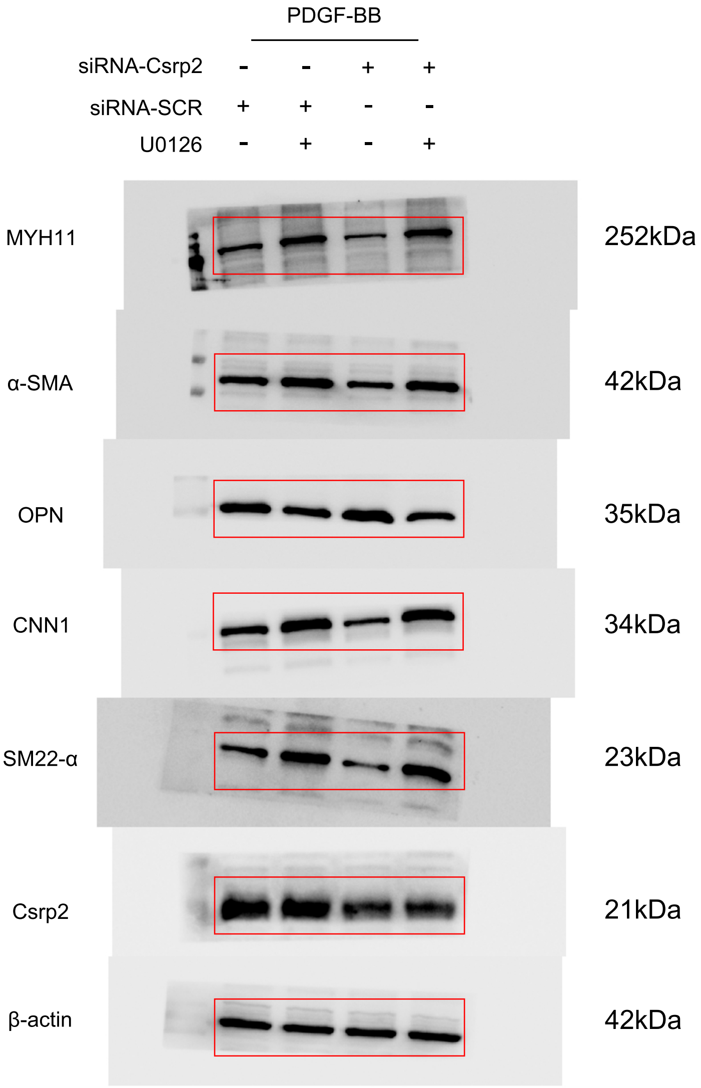

Raw blots in Figure 7.
